# Comparisons of quality of life between patients underwent peritoneal dialysis and hemodialysis: a systematic review and meta-analysis

**DOI:** 10.1186/s12955-020-01449-2

**Published:** 2020-06-18

**Authors:** Anan Chuasuwan, Siriporn Pooripussarakul, Ammarin Thakkinstian, Atiporn Ingsathit, Oraluck Pattanaprateep

**Affiliations:** 1 Department of Clinical Epidemiology and Biostatistics, Faculty of Medicine, Ramathibodi Hospital, Mahidol University, 270 Rama VI Rd., Ratchathewi, Bangkok, Thailand; 2 Nephrology Division, Department of Medicine, Bhumibol Adulyadej Hospital, Bangkok, Thailand; 3 Nephrology Division, Department of Medicine, Faculty of Medicine, Ramathibodi Hospital, Mahidol University, Bangkok, Thailand

**Keywords:** EQ-5D, Hemodialysis, KDQOL, Peritoneal dialysis, Health-related quality of life

## Abstract

**Objective:**

End-stage renal disease (ESRD) leads to renal replacement therapy and certainly has an impact on patients’ health-related quality of life (HRQoL). This study aimed to review and compare the HRQoL between peritoneal dialysis (PD) and hemodialysis (HD) patients using the 36-Item Short Form Health Survey (SF-36), EuroQoL-5-dimension (EQ-5D) and the Kidney Disease Quality of Life Instrument (KDQOL).

**Methodology:**

Systematic review was conducted by identify relevant studies through MEDLINE and SCOPUS up to April 2017. Studies were eligible with following criteria: studied in ESRD patients, compare any pair of renal replacement modalities, and reported HRQoL. The unstandardized mean differences (USMD) of HRQoL among modalities were calculated and pooled using a random-effect models if heterogeneity was present, otherwise a fixed-effect model was applied.

**Results:**

A total of twenty-one studies were included with 29,000 participants. Of them, mean age and percent male were 48.1 years and 45.1, respectively. The pooled USMD (95% CI) of SF-36 between PD and HD (base) were 1.86 (0.47, 3.24) and 0.42 (− 1.99, 2.82) for mental component and physical component summary scores, respectively. For EQ-5D, the pooled USMD of utility and visual analogue scale (VAS) score were 0.02 (− 0.06, 0.10) and 3.56 (1.73, 5.39), respectively. The pooled USMD of KDQOL were 9.67 (5.67, 13.68), 6.71 (− 5.92, 19.32) 6.30 (− 0.41, 12.18), 2.35 (− 4.35, 9.04), 2.10 (0.07, 4.13), and 1.21 (− 2.98, 5.40) for burden of kidney disease, work status, effects of kidney disease, quality of social interaction, symptoms, and cognitive function.

**Conclusion:**

Patients with chronic kidney disease (CKD) stage 5 or ESRD treated with PD had better generic HRQoL measured by SF-36 and EQ-5D than HD patients. In addition, PD had higher specific HRQoL by KDQOL than HD patients in subdomain of physical functioning, role limitations due to emotional problems, effects and burden of kidney disease.

## Introduction

End-stage renal disease (ESRD) can be treated by renal replacement therapy (RRT), which certainly impact on health-related quality of life (HRQoL) of the patients [1–3]. Actually, both ESRD treated by RRT or conservative management will have limitation in HRQoL, and also have increase in morbidity and mortality [4–7].

A goal of RRT in ESRD patients is not only improving patient’s survival but also achieving in well-being [8–12]. The various modalities of RRT which included hemodialysis (HD), peritoneal dialysis (PD), and kidney transplantation (KT), have distinct advantages and disadvantages. HD continues to be the most common form of dialysis therapy in nearly all countries which contribute about 80%, follow by PD and KT [13]. Previous studies have demonstrated that dialysis patients have poorer HRQoL than general population [14, 15]. Thus, HRQoL is becoming more important outcome for ESRD and RRT [4, 12, 16, 17]. Assessment of HRQoL was subjective involving multidimensional measurements including physical function, emotional function, social function and treatment effectiveness from patients [8, 18]. Many instruments have been used to assess HRQoL including generic and disease-specific instruments. The generic instruments (e.g., 36-Item Short Form Health Survey (SF-36), European Quality of Life-5 Dimensions (EQ-5D), the World Health Organization Quality of Life-BREF (WHOQOL-BREF), 15-dimensional Health-related quality of life (15D-HRQoL), 12-item General Health Questionnaire (GHQ-12)) measure overall health and functions whereas the disease-specific instrument is used to focus on individual symptoms of a specific disease (e.g., Kidney Disease Quality of Life (KDQOL), Quality of Life Index-Dialysis, and Transplantation). Nevertheless, there is no consensus about standard instrument to measure HRQoL [2].

Most commonly used instruments for generic HRQoL are SF-36 and EQ-5D which have been used in either general patients or specific disease (e.g., chronic kidney disease, diabetes mellitus and hypertension) [8, 19–21]. The SF-36, consisted of 36-items, was introduced since 1993 as part of the Medical Outcomes Study (MOS) [22]. EQ-5D, firstly introduced by the EuroQOL Group in 1990, can be used in a wide range of health conditions and treatments [23] which is used for estimating preference weight for that health status. The disease specific instrument primarily used for ESRD was the KDQOL which was introduced in 2002 [12]. It is a self-report measure that includes a short form (SF) item health survey as the generic core and the multi-item scales targeted on kidney disease and dialysis, including burden of kidney disease, symptoms and problems with kidney disease, and effects of kidney disease.

Previous studies showed that KT patients generally have better HRQoL than dialysis patients [24–26]. Many studies had also compared HRQoL between PD and HD but the results were still controversial and inconclusive [27–29]. This might be due to different health care system and modalities of RRT, income, education, inadequate sample size, multicultural environments, psychological problems, severity of condition, instrument’s responsiveness, timing of follow-up and various instrument [3, 18, 30]. We hypothesize that RRT modality of PD, HD, and KT had different impact on HRQoL of ESRD patients. Therefore, we conducted this study to pool mean difference of HRQoL between PD, HD, and KT in CKD stage 5 or ESRD patients using data from observational studies.

## Methodology

This systematic review was conducted following the Preferred Reporting Items for Systematic Reviews and Meta-Analyses (PRISMA) Statement guideline [31] and registered at PROSPERO (number CRD42016048574).

### Search strategy

Literature searches through two major medical databases were performed independently by two researchers (AC and SP), i.e., MEDLINE via PubMed and SCOPUS using search strategies presented in Additional file 1. Any type of observational studies (e.g., cohort, case-control, or cross sectional study) published in English since inception through April 2017 were identified. Additional studies were identified through the reference lists of identified articles.4.

### Study selection

Inclusion criteria were constructed based on patient (P), interventions (I), comparator (C), and outcomes (O) as follows: P: patients with chronic kidney disease (CKD) stage 5 or ESRD; I/C: had any pair of RRT modalities including PD, HD, KT, and conservative management (CM) and; O: had any type of HRQoL. The exclusion criteria were: 1) study patients were acute kidney injury (AKI), 2) duplicated reports and 3) insufficient data for pooling.

### Interventions and outcomes measurement

Interventions were RRTs including PD, HD, KT, and CM with regimens used according to original studies. The outcomes of interest were HRQoLs which could be measured as follows:

### SF-36

The SF-36 consists of eight subdomains and 36 questions including 10 items of physical functioning (PF), 4 items of role limitations due to physical health (RP), 2 items of pain (P), 5 items of general health (GH), 4 items of energy (E), 2 items of social functioning (SF), 3 items of role limitations due to emotional problems (RE), and 5 items of emotional well-being (EW) [32]. Each domain is transformed into a 0 to 100 range on the assumption that each question carries equal weight. The lower score indicates the more disability, whereas the higher score indicates the more favorable health state, for example, a score of zero is equivalent to maximum disability and a score of 100 is equivalent to no disability. Then, average subdomain score was calculated by dividing total subdomain scores with a total numbers of item of that subdomain. In addition, two component summary scores are also used to illustrate physical component summary score (PCS = PF + RP + P + GH) and mental component summary score (MCS = E + SF + RE + EW) [33, 34].

### EQ-5D

The EQ-5D consists of a descriptive system and visual analogue scale (VAS) [23]. The descriptive system comprises five dimensions, i.e., mobility, self-care, usual activities, pain/discomfort, and anxiety/depression. Each dimension was graded as no problems, some/moderate problems, and severe/ extreme problems; which is known as three level version (EQ-5D-3L). The scores on these five dimensions can be presented as a health profile or can be converted to a single summary index number (utility). Health utility values range from 0 (death) to 1 (perfect health) but the values less than 0 are possible, and represent health states considered worse than death. The EQ VAS measured the patient’s self-rated health on a vertical visual analogue scale of 0 to 100 [23, 30].

### KDQOL

The KDQOL™ assessed both generic and kidney disease targeted quality of life originally had 134 items [35]. The short KDQOL (KDQOL-SF™), currently version 1.3 [36], consists of 36-item of SF-36 and 11 domains of kidney-disease-targeted domain including symptoms/problems (12 items), effects of kidney disease on daily life (8 items), burden of kidney disease (4 items), work status (2 items), cognitive function (3 items), quality of social interaction (3 items), sexual function (2 items), sleep (4 items). It also included multi-item measures of social support (2 items), dialysis staff encouragement (2 items) and patient satisfaction (1 item) [2, 3]. The shorter KDQOL 36-Item Survey (KDQOL-36™) consists of 12-item short form (SF-12) for generic chronic disease domain and 24 items for kidney-disease-targeted domain. The kidney disease-targeted domain focus on health-related concerns include symptoms/problems (12 items), effects of kidney disease on daily life (8 items), and burden of kidney disease (4 items) [12]. Each of these scales is scored by transforming into 0–100 scores and averaging across the items on each subdomain to create subdomain scores, the higher score indicates better HRQoL [37]. Our study focused on only 6 out of 11 subdomains (i.e., including symptoms, effects of kidney disease, burden of kidney disease, work status, cognitive function, and quality of social interaction) because they were most relevant to symptoms specific for a kidney disease and treatment managements whereas other items were overlapped with generic HRQoL of SF-36/12.

### Data extraction

Two authors (AC and SP) independently extracted data from the included studies. Disagreements were solved by discussion and adjudication with a third party (AT and AI). For each included articles, information was extracted regarding general characteristics of studies and patients, modalities of RRT, type of HRQoL and measurements. In addition, data for main pooling were extracted including number of subjects, mean along with standard deviation (SD) of HRQoL by RRT modality type. Firstly, we planned to compare any pair of all management option of ESRD patients (PD, HD, KT and CM), and any type of HRQoL instruments (SF-36, EQ-5D, KDQOL, WHOQOL-BREF, 15D-HRQoL and GHQ-12). Because of the limitation of available data, we finally selected the comparison between PD and HD in SF-36, EQ-5D and KDQOL.

### Quality assessment

Risk of bias was assessed using the Newcastle-Ottawa Scale (NOS) for cohort studies [38] and an adapted form of NOS for cross-sectional studies [39]. Two authors (AC and SP) independently assessed the quality of included studies, and disagreements were resolved by consensus. See supplement Table 1.

### Statistical analysis

Unstandardized mean difference (USMD) of HRQoL between PD and HD groups along with its 95% CI were calculated for each study. A pairwise meta-analysis was carried out to pool USMD across studies using a random effect models if heterogeneity was present, otherwise a fixed-effect model was applied. Heterogeneity was assessed by Cochrane’s Q test and a degree of heterogeneity was quantified using *I*^*2*^ statistic. If heterogeneity was present (*p-value* < 0.1 or *I*^*2* ≥^ 25%), sources of heterogeneity were explored using subgroup analysis by mean age groups, male percentage, and gross domestic product (GDP) classification. Publication bias was assessed by performing a funnel plot and Egger’s test. If the publication bias was assumed to exist, contour-enhanced funnel plots were used to distinguish the cause of asymmetry, for example, heterogeneity, selection bias. All analyses were performed using STATA® version 15.0 (STATA Corp, College Station, TX). *P* value less than 0.05 was considered as statistical significance, except heterogeneity test where *P* < 0.1 was used.

## Results

### Characteristic of included studies

**Fig. 1:**
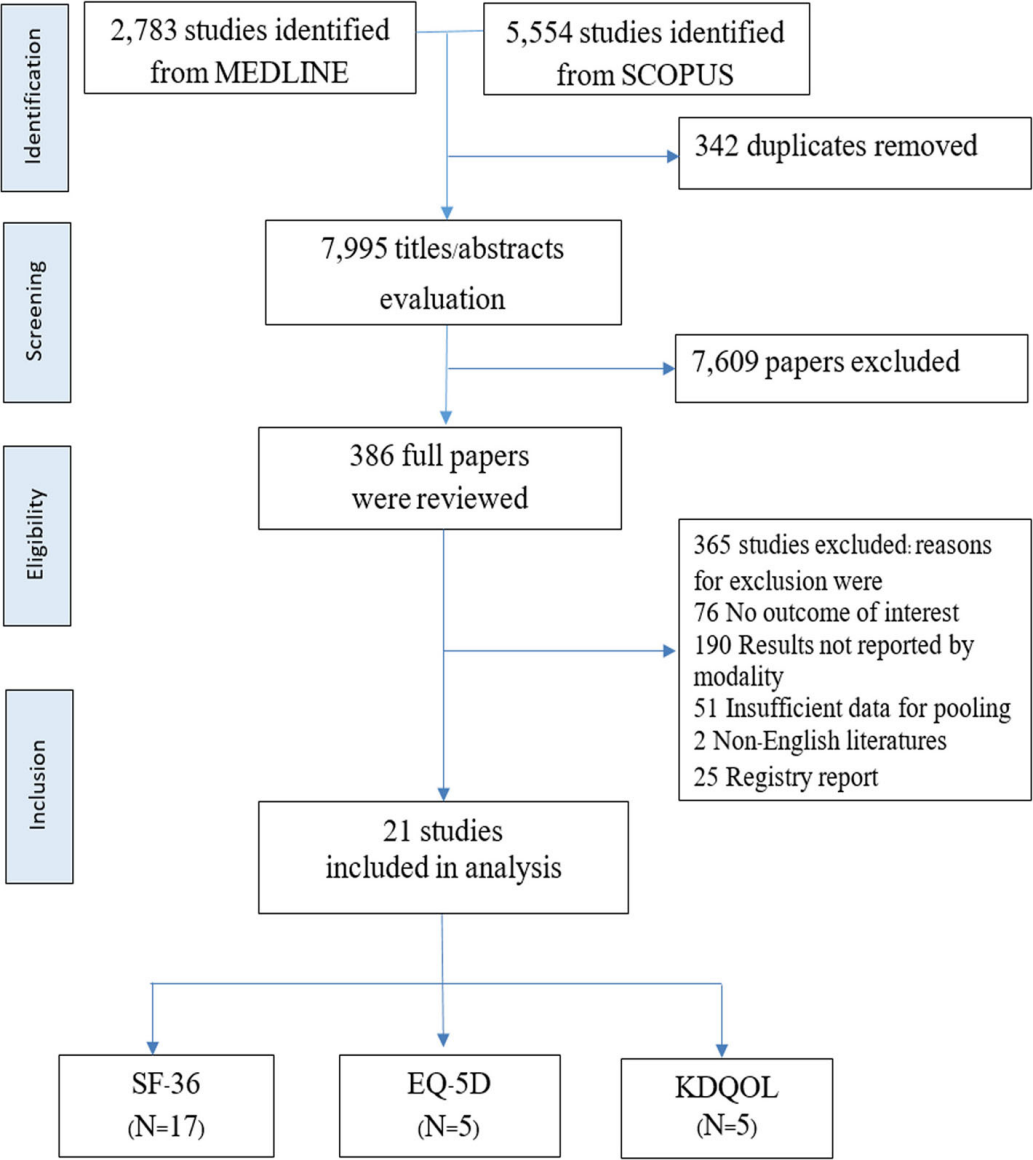
Flow chart of study selection

A total of 21 [2, 4, 10, 14, 15, 18, 26, 40–53] out of 7995 studies published between 1997 and 2016 were eligible, see Fig. 1. The characteristics of the included studies were shown in Table 1. These studies included around 29,000 participants (6035 PD and 22,967 HD) with mean age of 48.10 years and 45.10% of male. The study sample size ranged from 69 to 19,275 participants. Fifteen studies (71%) were conducted in high income countries in North America, Europe and Asia. Nineteen out of 21 studies (90%) were cross-sectional study, 17 and 5 studies assessed generic HRQoL by SF-36 and EQ-5D, whereas other 5 studies assessed KDQOL.

**Table 1:** Characteristics of included studies

Author (Year)	Country	GDP classification^a^	Study design	n	Mean age	% Male	HRQoL tool		
							SF-36	EQ5D	KDQOL
Merkus (1997) [15]	Netherlands	high income	CS	226	56.02	60.75	Y		
Wight (1998) [40]	UK	high income	CS	437	N/A	59.27	Y		
Merkus (1999) [10]	Netherlands	high income	Pro	139	56.83	60.54	Y		
Blake (2000) [41]	Ireland	high income	CS	112	44.03	70.54	Y		
Diaz-Buxo (2000) [42]	USA	high income	CS	19,275	58.66	51.61	Y		
Harris (2002) [43]	UK	high income	Pro	174	76.91	66.09	Y		
Wasserfallen (2004) [44]	USA	high income	CS	506	63.60	62.21		Y	
Kutner (2005) [45]	USA	high income	CS	3302	59.01	N/A	Y		Y
Lee (2005) [26]	UK	high income	CS	382	57.09	58.59	Y	Y	Y
Kalender (2007) [4]	Turkey	upper-middle	CS	141	49.83	57.50	Y		
Zhang (2007) [46]	China	upper-middle	CS	1062	58.90	49.44	Y		
Sayin (2007) [47]	Turkey	upper-middle	CS	136	44.66	67.75	Y		
Borowiak (2009) [48]	Poland	high income	CS	100	59.25	48.00		Y	
Kontodimopoulos (2009) [18]	Greece	high income	CS	874	55.39	59.84	Y		
Ibrahim (2011) [49]	Malaysia	upper-middle	CS	274	N/A	51.50	Y		
Turkmen (2012) [50]	Turkey	upper-middle	CS	154	53.92	55.84	Y		
Okpechi (2013) [51]	South Africa	upper-middle	CS	82	37.80	N/A	Y		Y
Czyzewski (2014) [52]	Poland	high income	CS	117	N/A	N/A	Y		Y
Yang (2015) [14]	Singapore	high income	CS	502	57.10	52.40		Y	
Kostro (2016) [2]	Poland	high income	CS	69	46.46	36.23	Y		Y
Chang (2016) [53]	Taiwan	high income	CS	1687	55.35	N/A		Y	

*Abbreviation*s: *GDP* Gross domestic product, *n* Number of participants, *HRQoL* Health-related quality of life, *SF-36* Short Form-36, *EQ-5D* EuroQol – 5dimension, *KDQOL* Kidney Disease Quality of Life Instrument, *N/A* Not available, *CS* Cross sectional, *Pro* Prospective, *Y* Yes; ^a^ GDP per capita were classified by the World Bank in 2016

### SF-36

**Fig. 2:**
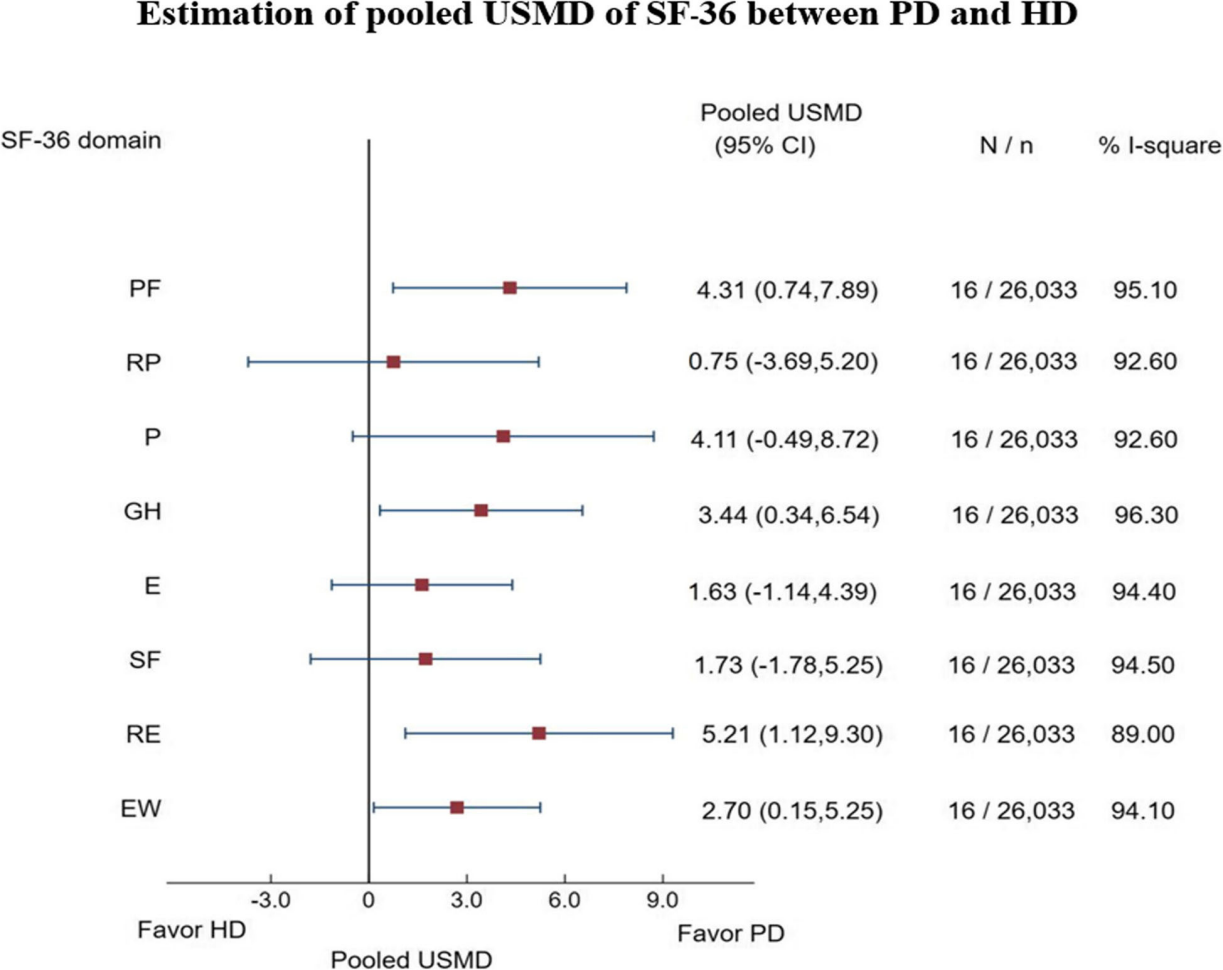
Estimation of pooled USMD of SF-36 between PD and HD

Data of 17 studies [2, 4, 10, 15, 18, 26, 40–43, 45–47, 49–52] were pooled for SF-36 subdomain (12 studies of SF-36 [4, 10, 15, 18, 40–43, 46, 47, 49, 50] and 5 studies [2, 26, 45, 51, 52] of KDQOL) and component summary scores. Mean (SD) subdomain scores by PD and HD modalities were described, see Supplement Table 2. Among 17 studies, one study [43] did not provide subdomain scores. USMDs between PD vs. HD were calculated for each subdomain and they were highly varied across studies with degree of heterogeneity *I*^*2*^ ranged from 89.0 to 96.3%, see Fig. 2. The USMDs of physical functioning, general health, role limitations due to emotional problems, and emotional well-being were significantly higher in PD than HD with the pooled USMDs (95% CI) of 4.31 (0.74, 7.89), 3.44 (0.34, 6.54), 5.21 (1.12, 9.30), and 2.70 (0.15, 5.25); whereas other subdomains were not significant, see Fig. 2 and Supplement Table 2. In addition, the pooled USMD of MCS was about 1.86 (0.47, 3.24) significantly higher in PD than HD but not for PDS [USMD = 0.42 (− 1.99, 2.82)], see Supplement Table 2.

Sources of heterogeneity were explored by subgroup analysis of mean age groups, percentage of male, and GDP classifications but none of them could explain the source of heterogeneity, except percent male > 60% for role limitations due to emotional problems yielded decrease of *I*^*2*^*t*o 27.90%, see Supplement Table 3. In addition, USMD of RE and EF for patients aged 55 years or older 5.54 (1.03, 10.05) and 3.72 (0.30, 7.14) significantly higher in PD than HD. Furthermore, the USMD of PF, P, and GH were about 6.29 (0.81, 11.77), 6.84 (3.12, 10.56), 5.22 (0.51, 9.92) significantly higher in PD than HD for patients who lived in upper-middle income whereas RF and EW were about 6.84 (2.36, 11.32) and 3.24 (0.38, 6.10) significantly higher in PD than HD in high income countries.

Egger’s test was applied for assessing publication bias indicating no evidence of asymmetry of the funnel, see Supplement Table 4, whereas funnel plots contrastingly showed asymmetrical for every subdomain, see Supplement Figure 1. The contour enhanced funnel plot demonstrated that some studies fell in both significant and non-significant areas, see Supplement Figure 2. These implied that the asymmetry of funnels might be due to heterogeneity than publication bias. For PCS and MCS, the funnel plot showed asymmetrical but Egger’s test failed to conclude that the funnel plot was asymmetry as follow: PCS (t statistics = − 0.69, *p* = 0.60) and MCS (t statistics = − 0.36, *p* = 0.75), see Supplement Table 4 and Supplement Figure 1. The contour enhanced funnel plot demonstrated that some studies fell in both significant and non-significant areas. These implied that the asymmetry of plot was due to heterogeneity than publication bias, see Supplement Figure 2.

### EQ-5D

**Fig. 3:**
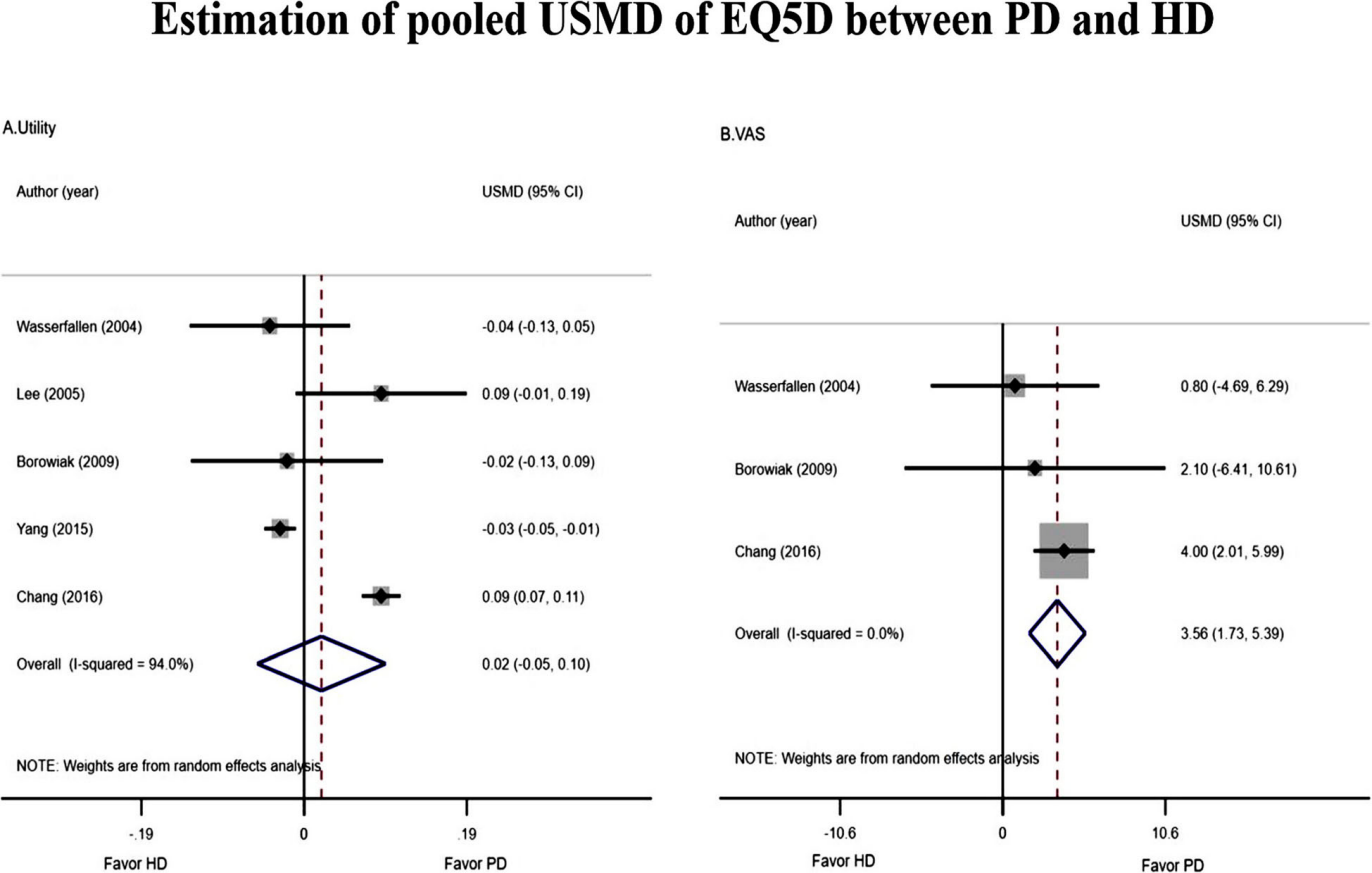
Estimation of pooled USMD of EQ5D between PD and HD

Data of mean EQ-5D scores of five studies [14, 26, 44, 48, 53] are described in Supplement Table 5. The USMD of utility between PD vs. HD was highly heterogeneous (*I*^*2*^ = 94.00%) but not for the VAS score (*I*^*2*^ = 0%). The pooled USMDs (95% CI) of utility and VAS score were 0.02 (− 0.06, 0.10) and 3.56 (1.73, 5.39), respectively, see Fig. 3 and Supplement Table 5. This could be interpreted that VAS score was about 3 units significantly higher in PD than HD.

The funnel plot showed little asymmetrical for Utility but not for VAS, see Supplement Figure 3, which corresponded with Egger’s test, see Supplement Table 4.

### KDQOL

**Fig. 4:**
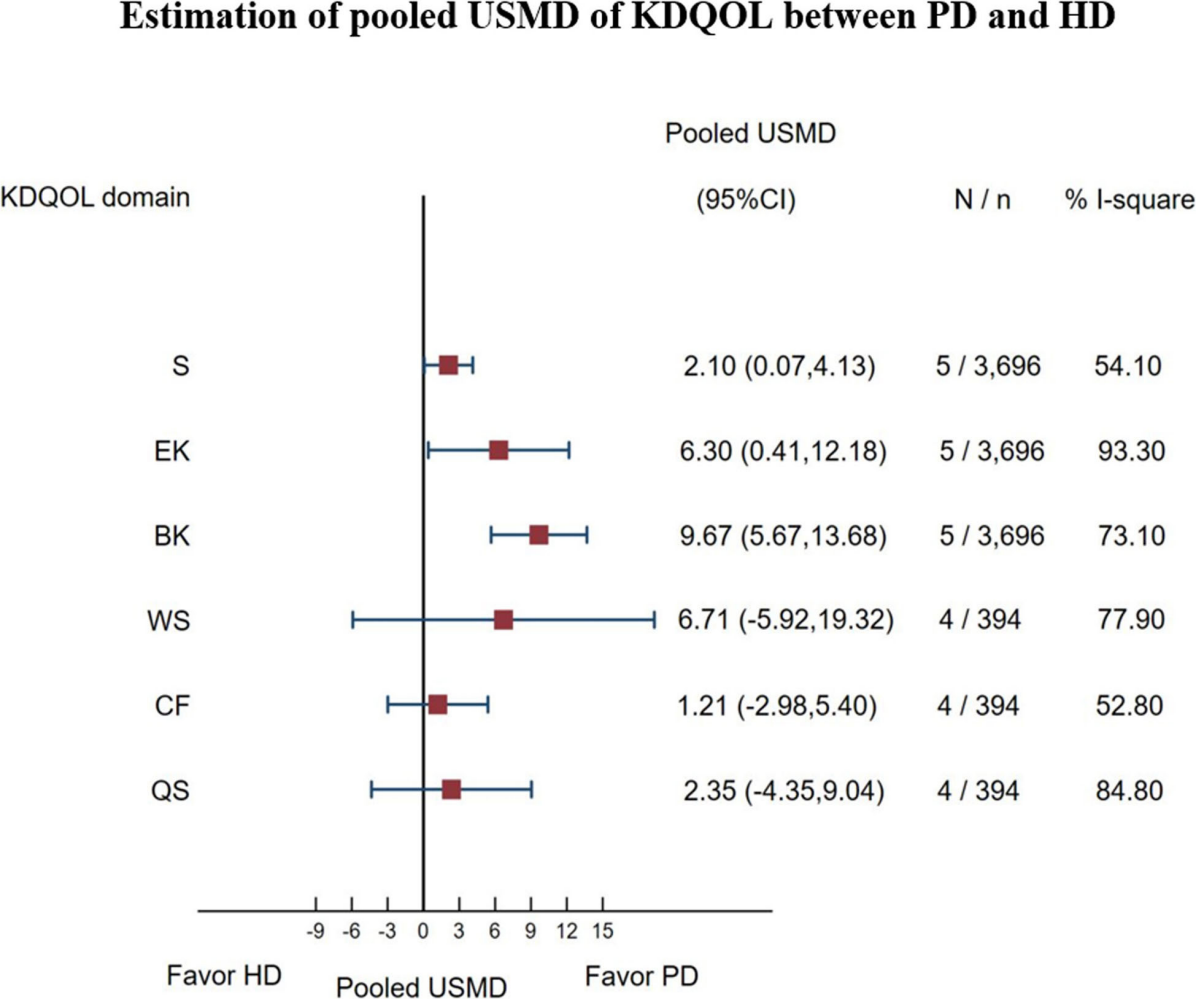
Estimation of pooled USMD of KDQOL between PD and HD

There were five studies [2, 26, 45, 51, 52] using KDQOL included for analysis, see Supplement Table 6. Six out of eleven subdomains were selected to analyze because they were most relevant to symptoms specific for a kidney disease or on its treatment management. USMDs for each subdomain between PD vs. HD were moderately to highly heterogeneous with the *I*^*2*^ ranged from 52.8 to 93.3%, see Fig. 4 and Supplement Table 6. The pooled USMDs (95% CI) were 2.10 (0.07, 4.13); 6.30 (− 0.41, 12.18), 9.67 (5.67, 13.68), 6.71 (− 5.92, 19.32), 1.21 (− 2.98, 5.40), 2.35 (− 4.35, 9.04) for symptoms, effects of kidney disease, burden of kidney disease, work status, cognitive function, and quality of social interaction, respectively, see Fig. 4 and Supplement Table 6.

The funnel plot showed little asymmetrical for every subdomain, see Supplement Figure 4, corresponded with the Egger’s tests, see Supplement Table 4.

## Discussion

This systematic review and meta-analysis showed the statistically higher mean scores of PF, GH, RE, E and MCS in SF-36, VAS in EQ-5D, and symptom, effects of kidney disease, and burden of kidney disease in KDQOL in PD than HD with effect sizes of mean differences of about 2 to 9 unit scores. Among these, only PF and RE in SF-36, and effects of kidney disease and burden of kidney disease in KDQOL may be clinically significant.

The minimal important difference (MID), the smallest difference that reflects a clinically meaningful [54–56], is used to interpret clinically significant changes in patient-reported outcome. The USMD of less than 4, 4–10, and more than 10 points are considered as small, moderate, and large effects [54, 57]. As for our pooling SF-36, only subdomain scores of PF and RE were clinically significant higher in PD than HD. The MCS was also higher in PD than HD but this did not reach to clinical significance.

In the kidney specific HRQoL, the pooled USMD showed small effects in favor of PD for symptoms, cognitive function and quality of social interaction but moderate effects in favor of PD were detected for effects of kidney disease, burden of kidney disease and work status. We found the USMD of quality of social interaction in KDQOL was approximately the same trend with SF subdomain in SF-36.

Source of heterogeneity was explored by performing subgroup analysis of SF-36 by age group (< 55, > 55 years), percentage of male (< 60, > 60%) and GDP classification (high-income, upper-middle income). Although a degree of heterogeneity could not identify, pooling HRQoLs within subgroups showed more benefit of PD than HD in some subgroups. For instance, RE and EW scores were higher in PD than HD in patients aged 55 years or older, i.e., PD patients had better emotion than the HD particularly in patients aged 55 years or older; this might be due to PD was mostly be performed at home or comfortable place where the environment was more relax and would result in better emotion than the HD which was performed in center/hospital. With the GDP classification, the high-income group showed significantly higher USMD in RE and EW subdomain, while the upper-middle income group showed significantly higher USMD in PF, P and GH subdomain respectively. However, these subgroup results were still high heterogeneity.

Previous systematic review and meta-analysis [58] published in 2007 assessed generic HRQoL measured by MOS SF-36 including 52 studies published before June 2005. They found HD had higher mean score in RP and VT subdomains, whereas the rest subdomain (PF, BP, GH, SF, RE and MH) were predominant in PD. Results of our study were consistent that favor PD over HD.

A recent systematic review and meta-analysis [59] published in 2017 assessed specific HRQoL by KDQOL-SF or − 36 including 7 studies indicating higher effect of kidney disease in PD than HD patients, which was consistent with our finding. In additional to this, we also found that burden of kidney disease and symptoms were also higher in PD than HD.

The EQ-5D is the most frequently used health utility instrument for calculating quality-adjusted life-year (QALY) based on the actual measurement of patients’ HRQoL [60]. This study showed the same direction of EQ-5D to SF-36 and KDQOL that favor PD than HD. The USMD of Utility score larger than VAS and non-significant may explain by the limitation of response set of three levels, which may cause less sensitivity to capture the real difference in health, while the VAS was freely and directly score from the patient’s subjective feeling [61].

Our study had some strengths. We performed systematic review and meta-analysis compared HRQoL between PD and HD using the most frequently utility both generic (SF-36) and kidney specific QOL (KDQOL) instrument [62], and another common generic HRQoL, EQ-5D, can further incorporated into cost-effectiveness analysis or cost-utility analysis. We performed and presented each subdomain of the instrument, these will help to understanding the real individual affected subdomain that contribute to patient HRQoL and may provide useful information and an opportunity to find the solution for specific management plan. We used USMD and MID which were direct and easy to understand.

Our study had some limitations. First, the data were from observational studies mostly cross-sectional design, which may prone to selection and confounding biases. Thus, the result was the average differences at point of time without direction or trend. Then, this results had to interpret with caution. Some author suggested a prospective repeated-measures experimental design to assess the real differences in quality of life among RRT patients [63]. Furthermore, serial assessment may be a useful way to monitor disease course and response to therapy [64]. Moreover, HRQoL assessments had been shown to improve patient–physician communication [65]. However, the focus of this study was on HRQoL difference in PD and HD. Second, it was sometimes unclear whether the HRQoL superiority is due to real benefits of the dialysis treatment regardless of differences modality or modality specific therapy or preexisting non treatment difference between groups. We had limited to extract the demographic and clinical data that might have been associated with HRQoL, such as marital status, educational level, socioeconomic status, dialysis duration [66], remaining renal function and underlying kidney disease, comorbidity [48], adequacy of dialysis, hemoglobin level or other clinical parameters on the patients’ perceptions of HRQoL and mental health. And some issues that lead to bias, for example, issue of case-mix differences [63, 67]; dialysis patients with severe comorbid illnesses and could not give self-report were also excluded from the study; the bias of selection of dialysis modalities, as the PD usually offered to patient with good family support, less comorbidity and higher education, etcetera. Others factors may affect the HRQoL, for example, anemia [68] and dose of dialysis [69, 70]. And again we had no HRQoL data at the treatment initiation and at the end of the study period which would be analysed for the actual treatment effect at the time. To counter the differences baseline from selection bias, the trend of improvement may more important than the time point HRQoL. We considered only studies published in English, omitting non-English studies might result in selection bias. However, given a lot of English studies available for us to pooling RRT effects on HRQoL, including some of non-English studies should not change much results. Finally, this study had shown high heterogeneity in the pool results. We explored source of heterogeneity by subgroup analysis by mean age groups, male percentage, and GDP classification, but we could not find the source from this factors. Therefore, there may be other factors caused heterogeneity but working with summary data did not allow to identify the other specific cause/s of heterogeneity. Although we could identify benefit of PD over HD in higher HRQoL of RE and EW for patients aged 55 years or older and high income countries, estimation of USMDs were quite imprecise. We did not perform subgroup analysis in several important factor, such as comorbidities, duration of dialysis and etcetera, because of our inability to obtain these data. Publication bias was also explored but the results suggested to heterogeneity.

Because of HRQoL focuses specifically on the influence of health, illness, and medical treatment on HRQoL [64], in HD patient, there was a finding shown that lower scores on HRQoL were strongly associated with higher risk of death and hospitalization [17]. There may be HRQoL preferences for PD over HD, but the selection an appropriate dialysis for an individual patient should be made by considering all possible factors with the patient and their relatives. This is an important issue.

## Conclusion

This study showed patients with chronic kidney disease (CKD) stage 5 or ESRD treated with PD had better overall HRQoL than HD patients by using SF-36, EQ-5D and KDQOL self-report tools and had significantly moderately better in subdomain of physical functioning, role limitation due to emotional problem, effects and burden of kidney disease. Future studies should explore the trend of differences over time and the association to clinical outcome such as hospitalization and mortality.

## Supplementary information

**Additional file 1.**

**Additional file 2.**

**Additional file 3.**

## Data Availability

Not applicable.
