# Supplementary material for: Comparisons of quality of life between patients underwent peritoneal dialysis and hemodialysis: a systematic review and meta-analysis
Source: Health Qual Life Outcomes. 2020 Jun 18;18:191. doi: 10.1186/s12955-020-01449-2 (PMC7302145; doi:10.1186/s12955-020-01449-2)
Supplement: Supplementary file 1 — Additional file 1. [file 12955_2020_1449_MOESM1_ESM.docx]

**Appendix I. Search terms and strategies**

Search terms were constructed according to PICO model: patient (P), intervention (I), comparison (C) and outcome (O).

Patient

1. Chronic kidney disease stage 5, or stage V
2. End stage renal disease (MeSH)

Intervention / Comparison

- - - 1. Renal replacement therapy (MeSH)

1. Haemodialysis or Haemodiafiltration
2. Peritoneal dialysis (MeSH)
3. Kidney transplantation (MeSH)

Outcome

- - - 1. Death (MeSH)
      2. Cardiovascular event (MeSH)
      3. Cardiovascular disease (MeSH)
      4. Myocardial infarction (MeSH)
      5. Arrhythmia (MeSH)
      6. Ischemic heart Disease (MeSH)
      7. Heart failure (MeSH)
      8. Coronary artery disease (MeSH)
      9. Quality of life (MeSH)

Strategies for searching included the usage of ‘or’ operation to the search terms within the domain and using ‘and’ operation to the search terms between the domains.

The final search terms and strategy were as following:

(((((((End stage renal disease [MeSH Terms]) OR "chronic kidney disease stage 5") OR "chronic kidney disease stage V"))

AND

(((((Renal replacement therapy [MeSH Terms]) OR Peritoneal dialysis [MeSH Terms]) OR haemodialysis) OR haemodiafiltration) OR kidney transplant [MeSH Terms]))

AND

((((((((death [MeSH Terms]) OR (("cardiovascular diseases" [MeSH Terms]) OR "cardiovascular event")) OR "myocardial infarction" [MeSH Terms]) OR "severe arrhythmia") OR "ischemic heart disease") OR "heart failure" [MeSH Terms]) OR "coronary artery disease" [MeSH Terms]) OR "quality of life" [MeSH Terms])))

AND

((("randomized controlled trials as topic" [MeSH Terms]) OR "cohort studies" [MeSH Terms]) OR "cross sectional study")

*Study selection*

Any type of original research, which study design could be

- Randomized control trial (RCT)

- Observational study including cohort and cross sectional study

- Study in English language

*Inclusion criteria*

1. Studied in patients with CKD stage 5 or ESRD
2. Compared between any pair of following treatment modalities: HD, PD, KT, CT
3. Had at least one of the following outcomes: all-cause/ cardiovascular mortality, major complications including cardiovascular events, infection and malignancy, quality of life

*Exclusion criteria*

1. Study patients were acute renal failure / acute kidney injury
2. Duplicated reports
3. Insufficient data for pooling

*Ineligible study coding*

1. Duplication articles
2. No comparison group
3. No relevant outcomes
4. No quantitative data collection
5. Systematic review with/without meta-analysis
6. Narrative review, letter to editor,
7. Results not reported by treatment modality

*Adding*

1. Bibliography Review
