# Supplementary material for: Comparisons of quality of life between patients underwent peritoneal dialysis and hemodialysis: a systematic review and meta-analysis
Source: Health Qual Life Outcomes. 2020 Jun 18;18:191. doi: 10.1186/s12955-020-01449-2 (PMC7302145; doi:10.1186/s12955-020-01449-2)
Supplement: Supplementary file 2 — Additional file 2. [file 12955_2020_1449_MOESM2_ESM.doc]

**Suplement Table**

|  |  | Page |
| --- | --- | --- |
| Table 1. | Risk of bias assessment by Newcastle Ottawa Scale (NOS) | 2 |
| Table 2. | Unstandardized mean difference of subdomain score for SF-36 | 3 |
| Table 3. | Subgroup analysis of SF-36 | 12 |
| Table 4. | Assessment of publication bias | 13 |
| Table 5. | Unstandardized mean difference of subdomain score for EQ5D | 14 |
| Table 6 |  | 15 |

Table 1 Risk of bias assessment for cohort by Newcastle Ottawa Scale (NOS)

| Author (Year) | Study Design | Selection (Max 5) | | | | Comparability (Max 2) | Outcome (Max 3) | | | |
| --- | --- | --- | --- | --- | --- | --- | --- | --- | --- | --- |
|  |  | Representativeness of the sample | Sample size | Non-respondents | Ascertainment of the exposure |  | Assessment of the outcome | | Statistical test | |
| Merkus (1997) [[15](#_ENREF_14)] | CS | * | * | * | * | * | * | | * | |
| Wight (1998) [[40](#_ENREF_14)] | CS | * | * | * | * | * | * | | * | |
| Blake (2000) [4[1](#_ENREF_14)] | CS | * | * | * | ** | * | * | | * | |
| Diaz-Buxo (2000) [[42](#_ENREF_14)] | CS |  | * |  | ** |  |  | | * | |
| Wasserfallen (2004) [[44](#_ENREF_14)] | CS | * | * | * | * | ** | * | | * | |
| Lee (2005) [[26](#_ENREF_14)] | CS | * | * |  | * | * | * | | * | |
| Kutner (2005) [[45](#_ENREF_14)] | CS | * | * | * | * | ** | * | | * | |
| Kalender (2007) [[4](#_ENREF_14)] | CS | * | * |  | * | ** |  | | * | |
| Zhang (2007) [[46](#_ENREF_14)] | CS | * | * | * | * |  |  | | * | |
| Sayin (2007) [[47](#_ENREF_14)] | CS |  | * | * | ** | * | * | | * | |
| Borowiak (2009) [[48](#_ENREF_14)] | CS | * | * |  | ** | * | ** | | * | |
| Kontodimopoulos (2009) [[1](#_ENREF_14)8] | CS | * |  | * | ** | * |  | | * | |
| Ibrahim (2011) [[49](#_ENREF_14)] | CS |  | * | * | ** |  | * | |  | |
| Turkmen (2012) [[5](#_ENREF_14)0] | CS | * | * | * | ** | ** |  | | * | |
| Okpechi (2013) [[5](#_ENREF_14)1] | CS |  | * |  | ** | ** |  | | * | |
| Czyzewski (2014) [[5](#_ENREF_14)2] | CS |  | * |  | ** |  | * | | * | |
| Yang (2015) [1[4](#_ENREF_14)] | CS | * | * | * | ** | * | * | | * | |
| Kostro (2016) [[2](#_ENREF_14)] | CS |  |  | * | * | ** |  | | * | |
| Chang (2016) [[5](#_ENREF_14)3] | CS | * | * |  | * |  | * | | * | |
| Author (Year) | Study Design | Selection (Max 4) | | | | Comparability (Max 2) | Outcome (Max 3) | | | |
|  |  | Representativeness of the exposed cohort | Selection of the non exposed cohort | Ascertainment of exposure | Demonstration that outcome of interest was not present at start of study |  | Assessment of outcome | Was follow-up long enough for outomes to occur | | Adequacy of follow up of cohorts |
| Merkus (1999) [[1](#_ENREF_14)0] | Pro | * | * | * | * | * | * | * | | * |
| Harris (2002) [43] | Pro | * | * |  | ** | ** |  | * | | * |

CS, cross sectional; Pro, prospective.

Table 2. Describe mean subdomain score of SF-36 by RRT modalities

| Author (Year) | PD | | | HD | | | USMD | 95% CI | % weight |
| --- | --- | --- | --- | --- | --- | --- | --- | --- | --- |
|  | n | mean | sd | n | mean | sd |  |  |  |
| **Physical functioning** | | | | | | | | | |
| Merkus (1997) | 106 | 60.90 | 24.70 | 120 | 50.70 | 30.80 | 10.20 | 2.96, 17.44 | 6.08 |
| Wight (1998) | 109 | 40.60 | 23.70 | 100 | 33.60 | 24.50 | 7.00 | 0.45, 13.55 | 6.38 |
| Merkus (1999) | 55 | 48.80 | 2.50 | 84 | 53.40 | 2.10 | -4.60 | -5.40, -3.80 | 8.08 |
| Blake (2000) | 23 | 75.00 | 13.71 | 37 | 75.00 | 16.79 | 0.00 | -7.79, 7.79 | 5.86 |
| Diaz-Buxo (2000) | 2,520 | 44.82 | 28.71 | 16,755 | 41.40 | 30.00 | 3.42 | 2.21, 4.63 | 8.04 |
| Kutner (2005) | 1,623 | 47.31 | 28.11 | 1,679 | 37.96 | 28.91 | 9.35 | 7.41, 11.30 | 7.92 |
| Lee (2005) | 74 | 30.90 | 28.70 | 99 | 26.50 | 27.70 | 4.40 | -4.12, 12.92 | 5.55 |
| Zhang (2007) | 408 | 49.88 | 30.63 | 654 | 45.07 | 30.86 | 4.81 | 1.01, 8.61 | 7.43 |
| Kalender (2007) | 47 | 75.30 | 23.90 | 68 | 47.30 | 24.10 | 28.00 | 19.08, 36.92 | 5.39 |
| Sayin (2007) | 41 | 55.76 | 31.10 | 75 | 55.90 | 26.96 | -0.14 | -11.45, 11.17 | 4.48 |
| Kontodimopoulos (2009) | 65 | 49.20 | 30.70 | 642 | 49.20 | 30.60 | 0.00 | -7.83, 7.83 | 5.84 |
| Ibrahim (2011) | 91 | 75.28 | 17.31 | 183 | 72.57 | 18.15 | 2.71 | -1.72, 7.13 | 7.22 |
| Turkmen (2012) | 64 | 62.60 | 30.80 | 90 | 59.60 | 31.10 | 3.00 | -6.91, 12.91 | 5.00 |
| Okpechi (2013) | 26 | 67.50 | 5.40 | 56 | 65.40 | 7.10 | 2.10 | -0.69, 4.89 | 7.73 |
| Czyzewski (2014) | 30 | 52.14 | 18.58 | 40 | 49.78 | 25.37 | 2.36 | -7.94, 12.66 | 4.85 |
| Kostro (2016) | 25 | 55.00 | 25.00 | 44 | 58.00 | 25.00 | -3.00 | -15.27, 9.27 | 4.15 |
| Pooled USMD (*I^2^*= 95.10%) | | | | | | | 4.31 | 0.74, 7.89 | 100.00 |

PD, Peritoneal dialysis; HD, Hemodialysis, USMD, Unstandardized mean difference; 95%CI, 95% Confidence interval.

Table 2. Describe mean subdomain score of SF-36 by RRT modalities (con.)

| Author (Year) | PD | | | HD | | | USMD | 95% CI | % weight |
| --- | --- | --- | --- | --- | --- | --- | --- | --- | --- |
|  | n | mean | sd | n | mean | sd |  |  |  |
| **Role limitations due to physical health** | | | | | | | | | |
| Merkus (1997) | 55 | 29.40 | 5.40 | 84 | 39.10 | 4.50 | 3.10 | -6.77, 12.97 | 6.14 |
| Wight (1998) | 23 | 37.50 | 18.28 | 37 | 37.50 | 19.75 | -3.20 | -13.08, 6.68 | 6.13 |
| Merkus (1999) | 2,520 | 33.28 | 38.35 | 16,755 | 33.10 | 39.40 | -9.70 | -11.42, -7.98 | 8.69 |
| Blake (2000) | 1,623 | 26.59 | 36.79 | 1,679 | 21.06 | 34.03 | 0.00 | -9.81, 9.81 | 6.16 |
| Diaz-Buxo (2000) | 74 | 60.60 | 42.40 | 99 | 60.40 | 44.10 | 0.18 | -1.43, 1.79 | 8.70 |
| Kutner (2005) | 408 | 26.41 | 48.05 | 654 | 22.36 | 45.61 | 5.53 | 3.11, 7.95 | 8.58 |
| Lee (2005) | 47 | 69.20 | 38.40 | 68 | 32.70 | 42.30 | 0.20 | -12.79, 13.19 | 5.02 |
| Zhang (2007) | 41 | 39.10 | 42.06 | 75 | 40.69 | 38.41 | 4.05 | -1.78, 9.88 | 7.64 |
| Kalender (2007) | 65 | 30.90 | 40.00 | 642 | 40.30 | 44.40 | 36.50 | 21.61, 51.39 | 4.43 |
| Sayin (2007) | 91 | 76.79 | 21.62 | 183 | 68.44 | 19.78 | -1.59 | -17.12, 13.94 | 4.24 |
| Kontodimopoulos (2009) | 64 | 23.00 | 36.30 | 90 | 56.90 | 47.80 | -9.40 | -19.71, 0.91 | 5.97 |
| Ibrahim (2011) | 26 | 56.10 | 8.30 | 56 | 52.30 | 5.60 | 8.34 | 3.06, 13.63 | 7.83 |
| Turkmen (2012) | 30 | 35.71 | 42.42 | 40 | 30.00 | 40.48 | -33.90 | -47.19, -20.61 | 4.93 |
| Okpechi (2013) | 25 | 23.00 | 35.00 | 44 | 15.00 | 31.00 | 3.80 | 0.29, 7.31 | 8.34 |
| Czyzewski (2014) | 55 | 29.40 | 5.40 | 84 | 39.10 | 4.50 | 5.71 | -13.98, 25.40 | 3.23 |
| Kostro (2016) | 23 | 37.50 | 18.28 | 37 | 37.50 | 19.75 | 8.00 | -8.50, 24.50 | 3.98 |
| Pooled USMD (*I^2^*= 92.60%) | | | | | | | 0.75 | -3.69, 5.20 | 100.00 |

PD, Peritoneal dialysis; HD, Hemodialysis, USMD, Unstandardized mean difference; 95%CI, 95% Confidence interval.

Table 2. Describe mean subdomain score of SF-36 by RRT modalities (con.)

| Author (Year) | PD | | | HD | | | USMD | 95% CI | % weight |
| --- | --- | --- | --- | --- | --- | --- | --- | --- | --- |
|  | n | mean | sd | n | mean | sd |  |  |  |
| **Pain** | | | | | | | | | |
| Merkus (1997) | 106 | 74.20 | 23.20 | 120 | 63.70 | 27.30 | 10.50 | 3.92, 17.09 | 6.43 |
| Wight (1998) | 109 | 59.00 | 24.50 | 100 | 48.60 | 23.90 | 10.40 | 3.84, 16.97 | 6.44 |
| Merkus (1999) | 55 | 59.30 | 3.60 | 84 | 69.90 | 3.00 | -10.60 | -11.75, -9.45 | 7.37 |
| Blake (2000) | 23 | 84.00 | 14.26 | 37 | 100.00 | 19.75 | -16.00 | -24.63, -7.37 | 5.88 |
| Diaz-Buxo (2000) | 2,520 | 60.13 | 27.99 | 16,755 | 57.20 | 28.60 | 2.93 | 1.75, 4.10 | 7.37 |
| Kutner (2005) | 1,623 | 63.44 | 26.91 | 1,679 | 56.08 | 29.71 | 7.36 | 5.43, 9.29 | 7.31 |
| Lee (2005) | 74 | 52.70 | 13.70 | 99 | 50.70 | 12.20 | 2.00 | -1.94, 5.94 | 7.03 |
| Zhang (2007) | 408 | 52.16 | 26.21 | 654 | 43.02 | 22.92 | 9.14 | 6.05, 12.23 | 7.17 |
| Kalender (2007) | 47 | 74.60 | 28.30 | 68 | 57.70 | 26.40 | 16.90 | 6.66, 27.14 | 5.42 |
| Sayin (2007) | 41 | 65.74 | 31.46 | 75 | 62.84 | 29.27 | 2.90 | -8.79, 14.59 | 5.02 |
| Kontodimopoulos (2009) | 65 | 57.10 | 35.20 | 642 | 59.90 | 32.90 | -2.80 | -11.73, 6.13 | 5.79 |
| Ibrahim (2011) | 91 | 81.82 | 20.15 | 183 | 74.07 | 22.34 | 7.75 | 2.50, 13.00 | 6.76 |
| Turkmen (2012) | 64 | 77.60 | 26.80 | 90 | 74.30 | 30.70 | 3.30 | -5.83, 12.43 | 5.74 |
| Okpechi (2013) | 26 | 79.90 | 5.40 | 56 | 76.70 | 3.50 | 3.20 | 0.93, 5.47 | 7.28 |
| Czyzewski (2014) | 30 | 65.89 | 28.09 | 40 | 45.50 | 29.79 | 20.39 | 6.74, 34.04 | 4.49 |
| Kostro (2016) | 25 | 56.00 | 28.00 | 44 | 52.00 | 27.00 | 4.00 | -9.57, 17.57 | 4.51 |
| Pooled USMD (*I^2^*=97.00%) | | | | | | | 4.11 | -0.49, 8.72 | 100.00 |

PD, Peritoneal dialysis; HD, Hemodialysis, USMD, Unstandardized mean difference; 95%CI, 95% Confidence interval.

Table 2. Describe mean subdomain score of SF-36 by RRT modalities (con.)

| Author (Year) | PD | | | | | | HD | | | | | USMD | 95% CI | % weight |
| --- | --- | --- | --- | --- | --- | --- | --- | --- | --- | --- | --- | --- | --- | --- |
|  | n | | mean | | sd | | n | | mean | | sd |  |  |  |
| **General health** | | | | | | | | | | | | | | |
| Merkus (1997) | 106 | 46.40 | | 20.50 | | 120 | | 43.00 | | 20.20 | | 3.40 | -1.92, 8.72 | 6.29 |
| Wight (1998) | 109 | 35.10 | | 21.30 | | 100 | | 31.60 | | 21.40 | | 3.50 | -2.30, 9.30 | 6.08 |
| Merkus (1999) | 55 | 38.90 | | 2.30 | | 84 | | 43.70 | | 1.90 | | -4.80 | -5.53, -4.07 | 7.69 |
| Blake (2000) | 23 | 52.00 | | 15.54 | | 37 | | 46.00 | | 17.78 | | 6.00 | -2.55, 14.55 | 4.86 |
| Diaz-Buxo (2000) | 2,520 | 42.30 | | 22.08 | | 16,755 | | 43.70 | | 22.00 | | -1.40 | -2.32, -0.48 | 7.67 |
| Kutner (2005) | 1,623 | 41.66 | | 21.60 | | 1,679 | | 38.23 | | 20.75 | | 3.43 | 1.99, 4.88 | 7.59 |
| Lee (2005) | 74 | 29.30 | | 18.40 | | 99 | | 25.00 | | 19.30 | | 4.30 | -1.36, 9.96 | 6.14 |
| Zhang (2007) | 408 | 36.75 | | 21.72 | | 654 | | 27.65 | | 18.82 | | 9.10 | 6.55, 11.65 | 7.33 |
| Kalender (2007) | 47 | 55.70 | | 23.90 | | 68 | | 39.80 | | 20.30 | | 15.90 | 7.54, 24.27 | 4.94 |
| Sayin (2007) | 41 | 41.25 | | 23.60 | | 75 | | 39.96 | | 19.87 | | 1.29 | -7.22, 9.80 | 4.88 |
| Kontodimopoulos (2009) | 65 | 38.10 | | 22.80 | | 642 | | 36.30 | | 21.80 | | 1.80 | -3.99, 7.59 | 6.08 |
| Ibrahim (2011) | 91 | 64.40 | | 15.24 | | 183 | | 58.56 | | 15.54 | | 5.84 | 1.98, 9.70 | 6.89 |
| Turkmen (2012) | 64 | 34.70 | | 16.60 | | 90 | | 46.10 | | 23.70 | | -11.40 | -17.77, -5.04 | 5.82 |
| Okpechi (2013) | 26 | 65.20 | | 3.80 | | 56 | | 56.20 | | 3.10 | | 9.00 | 7.33, 10.67 | 7.55 |
| Czyzewski (2014) | 30 | 42.14 | | 19.29 | | 40 | | 28.00 | | 12.29 | | 14.14 | 6.26, 22.02 | 5.15 |
| Kostro (2016) | 25 | 32.00 | | 15.00 | | 44 | | 34.00 | | 19.00 | | -2.00 | -10.13, 6.13 | 5.04 |
| Pooled USMD (*I^2^*= 96.30%) | | | | | | | | | | | | 3.44 | 0.34, 6.54 | 100.00 |

PD, Peritoneal dialysis; HD, Hemodialysis, USMD, Unstandardized mean difference; 95%CI, 95% Confidence interval.

Table 2. Describe mean subdomain score of SF-36 by RRT modalities (con.)

| Author (Year) | PD | | | HD | | | USMD | 95% CI | % weight |
| --- | --- | --- | --- | --- | --- | --- | --- | --- | --- |
|  | n | mean | sd | n | mean | sd |  |  |  |
| **Energy** | | | | | | | | | |
| Merkus (1997) | 106 | 51.60 | 17.90 | 120 | 48.90 | 24.00 | 2.70 | -2.78, 8.18 | 6.27 |
| Wight (1998) | 109 | 35.80 | 12.20 | 100 | 34.50 | 13.20 | 1.30 | -2.16, 4.76 | 7.36 |
| Merkus (1999) | 55 | 44.50 | 2.40 | 84 | 48.50 | 2.00 | -4.00 | -4.77, -3.24 | 8.26 |
| Blake (2000) | 23 | 60.00 | 16.45 | 37 | 47.50 | 16.79 | 12.50 | 3.87, 21.13 | 4.59 |
| Diaz-Buxo (2000) | 2,520 | 42.26 | 23.87 | 16,755 | 44.70 | 23.10 | -2.44 | -3.43, -1.44 | 8.23 |
| Kutner (2005) | 1,623 | 42.44 | 23.50 | 1,679 | 39.92 | 23.01 | 2.52 | 0.93, 4.11 | 8.09 |
| Lee (2005) | 74 | 33.10 | 18.80 | 99 | 32.20 | 17.70 | 0.90 | -4.62, 6.42 | 6.24 |
| Zhang (2007) | 408 | 45.40 | 24.30 | 654 | 36.67 | 22.58 | 8.73 | 5.81, 11.66 | 7.61 |
| Kalender (2007) | 47 | 65.90 | 22.80 | 68 | 52.70 | 23.90 | 13.20 | 4.55, 21.85 | 4.58 |
| Sayin (2007) | 41 | 51.66 | 27.00 | 75 | 56.20 | 22.15 | -4.54 | -14.21, 5.13 | 4.12 |
| Kontodimopoulos (2009) | 65 | 45.90 | 26.40 | 642 | 47.90 | 24.50 | -2.00 | -8.69, 4.69 | 5.59 |
| Ibrahim (2011) | 91 | 66.03 | 15.85 | 183 | 60.22 | 19.77 | 5.80 | 1.47, 10.14 | 6.90 |
| Turkmen (2012) | 64 | 40.20 | 23.70 | 90 | 60.10 | 23.40 | -19.90 | -27.46, -12.34 | 5.13 |
| Okpechi (2013) | 26 | 65.20 | 4.20 | 56 | 58.00 | 2.80 | 7.20 | 5.43, 8.97 | 8.04 |
| Czyzewski (2014) | 30 | 50.00 | 17.43 | 40 | 49.00 | 14.68 | 1.00 | -6.72, 8.72 | 5.04 |
| Kostro (2016) | 25 | 43.00 | 22.00 | 44 | 41.00 | 18.00 | 2.00 | -8.13, 12.13 | 3.93 |
| Pooled USMD (*I^2^*= 94.40%) | | | | | | | 1.63 | -1.14, 4.39 | 100.00 |

PD, Peritoneal dialysis; HD, Hemodialysis, USMD, Unstandardized mean difference; 95%CI, 95% Confidence interval.

Table 2. Describe mean subdomain score of SF-36 by RRT modalities (con.)

| Author (Year) | PD | | | HD | | | USMD | 95% CI | % weight |
| --- | --- | --- | --- | --- | --- | --- | --- | --- | --- |
|  | n | mean | sd | n | mean | sd |  |  |  |
| **Social functioning** | | | | | | | | | |
| Merkus (1997) | 106 | 68.90 | 25.80 | 120 | 63.10 | 29.60 | 5.80 | -1.42, 3.02 | 6.11 |
| Wight (1998) | 109 | 50.00 | 28.80 | 100 | 41.90 | 23.20 | 8.10 | 1.04, 15.17 | 6.18 |
| Merkus (1999) | 55 | 61.30 | 3.30 | 84 | 67.90 | 2.70 | -6.60 | -7.65, -5.55 | 8.17 |
| Blake (2000) | 23 | 50.00 | 18.28 | 37 | 62.50 | 19.75 | -12.50 | -22.31, -2.69 | 5.02 |
| Diaz-Buxo (2000) | 2,520 | 65.21 | 28.49 | 16,755 | 64.10 | 29.40 | 1.11 | -0.09, 2.31 | 8.15 |
| Kutner (2005) | 1,623 | 59.30 | 27.48 | 1,679 | 53.58 | 29.83 | 5.72 | 3.77, 7.68 | 8.03 |
| Lee (2005) | 74 | 46.50 | 30.90 | 99 | 36.70 | 28.70 | 9.80 | 0.77, 18.83 | 5.34 |
| Zhang (2007) | 408 | 56.71 | 25.02 | 654 | 48.89 | 25.20 | 7.82 | 4.72, 10.92 | 7.74 |
| Kalender (2007) | 47 | 75.00 | 28.30 | 68 | 58.70 | 20.20 | 16.30 | 6.89, 25.71 | 5.18 |
| Sayin (2007) | 41 | 56.32 | 32.49 | 75 | 62.62 | 26.01 | -6.30 | -17.86, 5.26 | 4.36 |
| Kontodimopoulos (2009) | 65 | 54.90 | 28.00 | 642 | 58.10 | 30.50 | -3.20 | -10.40, 4.00 | 6.12 |
| Ibrahim (2011) | 91 | 83.52 | 17.34 | 183 | 77.32 | 21.53 | 6.19 | 1.46, 10.93 | 7.16 |
| Turkmen (2012) | 64 | 63.70 | 23.40 | 90 | 83.10 | 25.70 | -19.40 | -27.21, -11.59 | 5.85 |
| Okpechi (2013) | 26 | 70.90 | 5.50 | 56 | 73.10 | 3.60 | -2.20 | -4.52, 0.12 | 7.95 |
| Czyzewski (2014) | 30 | 74.11 | 19.28 | 40 | 58.75 | 24.33 | 15.36 | 5.14, 25.58 | 4.86 |
| Kostro (2016) | 25 | 52.00 | 30.00 | 44 | 49.00 | 21.00 | 3.00 | -10.30, 16.30 | 3.78 |
| Pooled USMD (*I^2^*= 94.50%) | | | | | | | 1.73 | -1.78, 5.25 | 100.00 |

PD, Peritoneal dialysis; HD, Hemodialysis, USMD, Unstandardized mean difference; 95%CI, 95% Confidence interval.

Table 2. Describe mean subdomain score of SF-36 by RRT modalities (con.)

| Author (Year) | PD | | | HD | | | USMD | 95% CI | % weight |
| --- | --- | --- | --- | --- | --- | --- | --- | --- | --- |
|  | n | mean | sd | n | mean | sd |  |  |  |
| **Role limitations due to emotional problems** | | | | | | | | | |
| Merkus (1997) | 106 | 63.80 | 39.80 | 120 | 52.50 | 45.40 | 11.30 | 0.19, 22.41 | 5.56 |
| Wight (1998) | 109 | 55.50 | 32.60 | 100 | 31.00 | 36.80 | 24.50 | 15.04, 33.96 | 6.27 |
| Merkus (1999) | 55 | 57.00 | 6.00 | 84 | 57.00 | 5.00 | 0.00 | -1.91, 1.91 | 9.24 |
| Blake (2000) | 23 | 100.00 | 18.28 | 37 | 100.00 | 19.75 | 0.00 | -9.81, 9.81 | 6.11 |
| Diaz-Buxo (2000) | 2,520 | 58.39 | 43.90 | 16,755 | 53.00 | 44.30 | 5.39 | 3.55, 7.23 | 9.25 |
| Kutner (2005) | 1,623 | 55.45 | 44.00 | 1,679 | 45.05 | 44.76 | 10.40 | 7.37, 13.43 | 8.96 |
| Lee (2005) | 74 | 83.60 | 34.70 | 99 | 85.80 | 31.20 | -2.20 | -12.21, 7.81 | 6.03 |
| Zhang (2007) | 408 | 57.65 | 48.06 | 654 | 42.36 | 47.94 | 15.29 | 9.35, 21.23 | 7.87 |
| Kalender (2007) | 47 | 68.10 | 39.90 | 68 | 42.30 | 42.80 | 25.80 | 10.52, 41.08 | 4.07 |
| Sayin (2007) | 41 | 46.14 | 44.33 | 75 | 50.37 | 39.13 | -4.23 | -20.43, 11.97 | 3.81 |
| Kontodimopoulos (2009) | 65 | 43.60 | 47.00 | 642 | 49.90 | 45.20 | -6.30 | -18.25, 5.65 | 5.23 |
| Ibrahim (2011) | 91 | 80.95 | 22.85 | 183 | 74.95 | 21.74 | 6.00 | 0.35, 11.65 | 7.99 |
| Turkmen (2012) | 64 | 20.80 | 36.70 | 90 | 60.40 | 45.80 | -39.60 | -52.65, -26.55 | 4.81 |
| Okpechi (2013) | 26 | 68.80 | 8.00 | 56 | 67.50 | 5.60 | 1.30 | -2.11, 4.71 | 8.85 |
| Czyzewski (2014) | 30 | 73.81 | 35.03 | 40 | 46.67 | 42.16 | 27.14 | 9.03, 45.25 | 3.31 |
| Kostro (2016) | 25 | 45.00 | 46.00 | 44 | 30.00 | 39.00 | 15.00 | -6.40, 36.40 | 2.64 |
| Pooled USMD (*I^2^*= 89.00%) | | | | | | | 5.21 | 1.12, 9.30 | 100.00 |

PD, Peritoneal dialysis; HD, Hemodialysis, USMD, Unstandardized mean difference; 95%CI, 95% Confidence interval.

Table 2. Describe mean subdomain score of SF-36 by RRT modalities (con.)

| Author (Year) | PD | | | HD | | | USMD | 95% CI | % weight |
| --- | --- | --- | --- | --- | --- | --- | --- | --- | --- |
|  | n | mean | sd | n | mean | sd |  |  |  |
| **Emotional well-being** | | | | | | | | | |
| Merkus (1997) | 106 | 72.20 | 16.90 | 120 | 63.30 | 20.50 | 8.90 | 4.02, 13.78 | 6.45 |
| Wight (1998) | 109 | 65.90 | 18.30 | 100 | 60.00 | 18.30 | 5.90 | 0.93, 10.87 | 6.39 |
| Merkus (1999) | 55 | 65.50 | 2.30 | 84 | 68.20 | 1.90 | -2.70 | -3.43, -1.97 | 8.39 |
| Blake (2000) | 23 | 86.00 | 15.36 | 37 | 82.00 | 14.22 | 4.00 | -3.77, 11.77 | 4.73 |
| Diaz-Buxo (2000) | 2,520 | 69.73 | 20.37 | 16,755 | 68.70 | 21.20 | 1.03 | 0.17, 1.89 | 8.36 |
| Kutner (2005) | 1,623 | 69.66 | 19.19 | 1,679 | 63.91 | 22.32 | 5.75 | 4.33, 7.17 | 8.23 |
| Lee (2005) | 74 | 59.30 | 17.90 | 99 | 53.10 | 17.60 | 6.20 | 0.85, 11.55 | 6.15 |
| Zhang (2007) | 408 | 68.08 | 21.83 | 654 | 58.77 | 23.56 | 9.31 | 6.53, 12.09 | 7.67 |
| Kalender (2007) | 47 | 69.40 | 21.10 | 68 | 58.30 | 19.90 | 11.10 | 3.44, 18.77 | 4.79 |
| Sayin (2007) | 41 | 60.56 | 23.93 | 75 | 58.33 | 22.48 | 2.23 | -6.69, 11.15 | 4.15 |
| Kontodimopoulos (2009) | 65 | 53.00 | 26.10 | 642 | 55.10 | 22.70 | -2.10 | -8.68, 4.48 | 5.40 |
| Ibrahim (2011) | 91 | 78.02 | 17.38 | 183 | 71.04 | 19.44 | 6.98 | 2.44, 11.53 | 6.66 |
| Turkmen (2012) | 64 | 49.40 | 16.90 | 90 | 69.80 | 19.50 | -20.40 | -26.18, -14.62 | 5.89 |
| Okpechi (2013) | 26 | 75.00 | 4.60 | 56 | 74.60 | 2.80 | 0.40 | -1.51, 2.31 | 8.06 |
| Czyzewski (2014) | 30 | 68.29 | 8.94 | 40 | 61.20 | 20.75 | 7.09 | -0.09, 14.27 | 5.06 |
| Kostro (2016) | 25 | 44.00 | 22.00 | 44 | 45.00 | 18.00 | -1.00 | -11.13, 9.13 | 3.62 |
| Pooled USMD (*I^2^*= 94.10%) | | | | | | | 2.70 | 0.15, 5.25 | 100.00 |

PD, Peritoneal dialysis; HD, Hemodialysis, USMD, Unstandardized mean difference; 95%CI, 95% Confidence interval.

Table 2. Describe mean subdomain score of SF-36 by RRT modalities (con.)

| Author (Year) | PD | | | HD | | | USMD | 95% CI | % weight |
| --- | --- | --- | --- | --- | --- | --- | --- | --- | --- |
|  | n | mean | sd | n | mean | sd |  |  |  |
| **Physical summary component score** | | | | | | | | | |
| Harris (2002) | 78 | 32.00 | 5.19 | 96 | 31.60 | 5.02 | 0.40 | -1.13, 1.93 | 25.73 |
| Kutner (2005) | 1,623 | 33.72 | 10.19 | 1,679 | 31.34 | 9.71 | 2.38 | 1.70, 3.06 | 28.08 |
| Lee (2005) | 74 | 33.70 | 10.80 | 99 | 33.00 | 10.40 | 0.70 | -2.50, 3.90 | 19.01 |
| Yang (2015) | 266 | 37.08 | 6.21 | 236 | 38.88 | 6.00 | -1.80 | -2.87, -0.73 | 27.18 |
| Pooled USMD (I^2^= 93.00%) | | | | | | | 0.42 | -1.99, 2.82 | 100.00 |
| **Mental summary component score** | | | | | | | | | |
| Harris (2002) | 78 | 54.60 | 8.85 | 96 | 52.60 | 8.36 | 2.00 | -0.58, 4.58 | 17.01 |
| Kutner (2005) | 1,623 | 46.91 | 11.07 | 1,679 | 44.32 | 12.24 | 2.59 | 1.79, 3.39 | 36.53 |
| Lee (2005) | 74 | 47.60 | 8.10 | 99 | 44.70 | 9.20 | 2.90 | 0.31, 5.49 | 16.96 |
| Yang (2015) | 266 | 46.57 | 7.80 | 236 | 46.31 | 7.86 | 0.26 | -1.11, 1.63 | 29.50 |
| Pooled USMD (*I^2^*= 65.60%) | | | | | | | 1.86 | 0.47, 3.24 | 100.00 |

PD, Peritoneal dialysis; HD, Hemodialysis, USMD, Unstandardized mean difference; 95%CI, 95% Confidence interval.

Table 3. Subgroup analysis of SF-36

| Factor | PF | | RP | | P | | GH | | E | | SF | | RE | | EW | |
| --- | --- | --- | --- | --- | --- | --- | --- | --- | --- | --- | --- | --- | --- | --- | --- | --- |
|  | USMD  (95%CI) | *I^2^* | USMD  (95%CI) | *I^2^* | USMD  (95%CI) | *I^2^* | USMD  (95%CI) | *I^2^* | USMD  (95%CI) | *I^2^* | USMD  (95%CI) | *I^2^* | USMD  (95%CI) | *I^2^* | USMD  (95%CI) | *I^2^* |
| **Age group** |  |  |  |  |  |  |  |  |  |  |  |  |  |  |  |  |
| <55 years | 5.16  (-2.91,13.23) | 84.5 | 1.90  (-11.72,15.52) | 90.1 | 2.11  (-5.36,9.58) | 80.5 | 3.13  (-4.52,10.77) | 89.6 | 1.80  (-7.44,11.03) | 91.2 | -3.74  (-12.51,5.02) | 86.9 | -0.94  (-14.86,12.98) | 89.8 | -0.82  (-9.07,7.43) | 91.2 |
| ≥55 years | 3.82  (-1.43,9.07) | 97.7 | -0.79  (-6.61,5.03) | 95.3 | 2.65  (-4.33,9.62) | 98.6 | 2.06  (-1.55,5.67) | 96.9 | 0.85  (-2.19,3.89) | 94.7 | 2.61  (-2.38,7.60) | 97.1 | 5.54  (1.03,10.05) | 89.2 | 3.72  (0.30,7.14) | 96.7 |
| **% Male** |  |  |  |  |  |  |  |  |  |  |  |  |  |  |  |  |
| <60 | 5.40  (1.82,8.97) | 75.2 | 0.91  (-5.82,7.64) | 87.3 | 5.74  (2.67,8.82) | 72.2 | 2.81  (-1.57,7.18) | 91.7 | 0.95  (-3.58,5.48) | 91.7 | 3.22  (-1.40,7.85) | 87.5 | 4.85  (-3.12,12.83) | 90.6 | 2.05  (-2.64,6.74) | 92.4 |
| ≥60 | 1.02  (-6.45,8.50) | 83.0 | -3.14  (-10.93,4.65) | 71.5 | -3.52  (-15.30,8.27) | 93.3 | 0.78  (-5.31,6.87) | 82.0 | 1.21  (-5.66,8.08) | 84.5 | -4.58  (-11.49,2.33) | 76.2 | 1.20  (-3.26,5.66) | 27.9 | 2.90  (-4.07,9.86) | 87.9 |
| **GDP classification** |  |  |  |  |  |  |  |  |  |  |  |  |  |  |  |  |
| High- income | 3.10  (-1.52,7.71) | 96.5 | -0.72  (-5.92,4.48) | 92.8 | 2.36  (-3.80,8.52) | 97.9 | 2.05  (-0.86,4.96) | 93.8 | 0.44  (-1.88,2.75) | 88.1 | 2.30  (-2.14,6.74) | 95.4 | 6.84  (2.36,11.32) | 86.7 | 3.24  (0.38,6.10) | 94.0 |
| Upper- middle  income | 6.29  (0.81,11.77) | 83.7 | 2.95  (-6.65,12.54) | 90.5 | 6.84  (3.12,10.56) | 66.9 | 5.22  (0.51,9.92) | 88.8 | 2.43  (-3.45,8.30) | 91.2 | 0.66  (-6.53,7.85) | 92.7 | 1.21  (-9.85,12.27) | 92.7 | 1.60  (-5.64,8.84) | 94.8 |

Abbreviation: E, Energy; EW, Emotional well-being; GH, General health; P, Pain; PF, Physical functioning; RE, Role limitations due to emotional problems; RP, Role limitations due to physical health; SF, Social functioning; GDP, Gross domestic product; USMD, Unstandardized mean difference.

Table 4. Assessment of publication bias

| QOL instrument | Funnel plot | | Egger’s test | | | Contour test | |
| --- | --- | --- | --- | --- | --- | --- | --- |
|  | Symmetry | Asymmetry | coefficient | t statistic | *p-value* | Significant area | Non-significant area |
| **SF-36** | | | | | | | |
| Physical functioning |  | √ | 2.75 | 1.96 | 0.07 | √ | √ |
| Role limitations due to physical health |  | √ | 1.10 | 0.81 | 0.43 | √ | √ |
| Pain |  | √ | 2.97 | 1.44 | 0.17 | √ | √ |
| General health |  | √ | 3.43 | 0.27 | 0.06 | √ | √ |
| Energy |  | √ | 2.41 | 1.70 | 0.11 | √ | √ |
| Social functioning |  | √ | 1.93 | 1.28 | 0.22 | √ | √ |
| Role limitations due to emotional problems |  | √ | 0.55 | 0.47 | 0.64 | √ | √ |
| Emotional well-being |  | √ | 1.95 | 1.39 | 0.19 | √ | √ |
| Physical component summary |  | √ | -3.19 | -0.69 | 0.60 | √ | √ |
| Mental component summary |  | √ | -0.80 | -0.36 | 0.75 | √ | √ |
| **EQ-5D** | | | | | | | |
| Utility |  | √ | 0.39 | 0.11 | 0.92 | √ | √ |
| VAS | √ |  | -1.04 | -1.62 | 0.35 | √ | √ |
| **KDQOL** | | | | | | | |
| Symptoms |  | √ | 1.11 | 1.07 | 0.36 | √ | √ |
| Effects of kidney disease |  | √ | -0.36 | -0.11 | 0.92 | √ | √ |
| Burden of kidney disease |  | √ | 1.15 | 0.73 | 0.52 | √ | √ |
| Work status |  | √ | -2.60 | -2.75 | 0.11 | √ | √ |
| Cognitive function |  | √ | -0.47 | -0.32 | 0.78 | √ | √ |
| Quality of social interaction |  | √ | 2.38 | 0.95 | 0.44 | √ | √ |

SF-36, 36-Item Short Form Health Survey; EQ-5D, European Quality of Life-5 Dimensions; KDQOL, Kidney Disease Quality of Life

Table 5. Summary pooled USMD for EQ-5D

| Author (Year) | PD | | | HD | | | USMD | 95% CI | % weight |
| --- | --- | --- | --- | --- | --- | --- | --- | --- | --- |
|  | n | mean | sd | n | mean | sd |  |  |  |
| **Utility** | | | | | | | | | |
| Wasserfallen (2004) | 50 | 0.581 | 0.32 | 456 | 0.62 | 0.30 | -0.04 | -0.13, 0.05 | 17.92 |
| Lee (2005) | 74 | 0.53 | 0.34 | 99 | 0.44 | 0.32 | 0.09 | -0.01, 0.19 | 17.26 |
| Borowiak (2009) | 50 | 0.5 | 0.31 | 50 | 0.52 | 0.26 | -0.02 | -0.13, 0.09 | 15.98 |
| Yang (2015) | 266 | 0.591 | 0.10 | 236 | 0.62 | 0.11 | -0.03 | -0.05, -0.01 | 24.51 |
| Poled USMD (*I^2^*= 94.00%) | | | | | | | 0.02 | -0.06, 0.10 | 100.00 |
| **VAS** | | | | | | | | | |
| Wasserfallen (2004) | 50 | 61.20 | 18.90 | 456 | 60.40 | 18.00 | 0.80 | -4.69, 6.29 | 11.08 |
| Borowiak (2009) | 50 | 55.30 | 21.70 | 50 | 53.20 | 21.70 | 2.10 | -6.41, 10.61 | 4.62 |
| Chang (2016) | 284 | 69.40 | 15.70 | 1,403 | 65.40 | 15.20 | 4.00 | 2.01, 5.99 | 84.30 |
| Pooled USMD (*I^2^*= 0%) | | | | | | | 3.56 | 1.73, 5.39 | 100.00 |

PD, Peritoneal dialysis; HD, Hemodialysis, USMD, Unstandardized mean difference; 95%CI, 95% Confidence interval.

Table 6. Summary pooled USMD for KDQOL

| Author (Year) | PD | | | HD | | | USMD | 95% CI | % weight |
| --- | --- | --- | --- | --- | --- | --- | --- | --- | --- |
|  | n | mean | sd | n | mean | sd |  |  |  |
| **Symptoms** | | | | | | | | | |
| Kutner (2005) | 1,623 | 72.39 | 15.94 | 1,679 | 70.48 | 16.88 | 1.91 | 0.79, 3.03 | 4.45 |
| Lee (2005) | 74 | 69.20 | 18.20 | 99 | 65.20 | 19.30 | 4.00 | -1.63, 9.63 | 8.54 |
| Okpechi (2013) | 26 | 73.20 | 4.40 | 56 | 73.20 | 2.30 | 0.00 | -1.80, 1.80 | 41.91 |
| Czyzewski (2014) | 30 | 73.85 | 3.13 | 40 | 65.51 | 19.96 | 8.34 | 2.05, 14.63 | 34.89 |
| Kostro (2016) | 25 | 65.00 | 19.00 | 44 | 61.00 | 18.00 | 4.00 | -5.15, 13.15 | 10.21 |
| Pooled USMD (*I^2^*=54.10%) | | | | | | | 2.10 | 0.07, 4.13 | 100.00 |
| **Effects of kidney disease** | | | | | | | | | |
| Kutner (2005) | 1,623 | 67.37 | 20.46 | 1,679 | 57.90 | 22.79 | 9.47 | 7.99, 10.95 | 24.36 |
| Lee (2005) | 74 | 64.50 | 19.80 | 99 | 56.70 | 23.6 | 7.80 | 1.32, 14.28 | 19.04 |
| Okpechi (2013) | 26 | 67.40 | 4.90 | 56 | 67.60 | 2.6 | -0.20 | -2.20, 1.80 | 24.05 |
| Czyzewski (2014) | 30 | 65.11 | 12.77 | 40 | 53.97 | 19.64 | 11.14 | 3.53, 18.75 | 17.50 |
| Kostro (2016) | 25 | 56.00 | 21.00 | 44 | 52.00 | 16 | 4.00 | -5.49, 13.49 | 15.06 |
| Pooled USMD (*I^2^*= 93.30%) | | | | | | | 6.30 | 0.41, 12.18 | 100.00 |
| **Burden of kidney disease** | | | | | | | | | |
| Kutner (2005) | 1,623 | 48.56 | 28.62 | 1,679 | 39.55 | 28.71 | 9.01 | 7.05, 10.97 | 8.38 |
| Lee (2005) | 74 | 36.30 | 26.00 | 99 | 25.20 | 23.90 | 11.10 | 3.53, 18.67 | 11.42 |
| Okpechi (2013) | 26 | 57.10 | 6.20 | 56 | 51.00 | 3.60 | 6.10 | 3.54, 8.66 | 33.11 |
| Czyzewski (2014) | 30 | 54.33 | 20.63 | 40 | 30.00 | 20.79 | 24.33 | 14.53, 34.13 | 31.34 |
| Kostro (2016) | 25 | 38.00 | 27.00 | 44 | 35.00 | 20.00 | 3.00 | -9.12, 15.12 | 15.76 |
| Pooled USMD (*I^2^*= 73.10%) | | | | | | | 9.67 | 5.67, 13.68 | 100.00 |

PD, Peritoneal dialysis; HD, Hemodialysis, USMD, Unstandardized mean difference; 95%CI, 95% Confidence interval.

Table 6. Summary pooled USMD for KDQOL (con.)

| Author (Year) | PD | | | HD | | | USMD | 95% CI | % weight |
| --- | --- | --- | --- | --- | --- | --- | --- | --- | --- |
|  | n | mean | sd | n | mean | sd |  |  |  |
| **Work status** | | | | | | | | | |
| Lee (2005) | 74 | 28.20 | 39.40 | 99 | 20.0 | 31.3 | 8.20 | -2.69, 19.09 | 17.78 |
| Okpechi (2013) | 26 | 54.10 | 8.30 | 56 | 36.4 | 5.3 | 17.70 | 14.22, 21.18 | 19.08 |
| Czyzewski (2014) | 30 | 28.57 | 37.80 | 40 | 44.4 | 46.4 | -15.87 | -35.61, 3.87 | 34.86 |
| Kostro (2016) | 25 | 34.00 | 45.00 | 44 | 27.0 | 40.0 | 7.00 | -14.23, 28.23 | 28.27 |
| Pooled USMD (*I^2^*= 77.90%) | | | | | | | 6.71 | -5.92, 19.32 | 100.00 |
| **Cognitive function** | | | | | | | | | |
| Lee (2005) | 74 | 79.60 | 19.70 | 99 | 72.60 | 20.30 | 7.00 | 0.99, 13.01 | 24.65 |
| Okpechi (2013) | 26 | 79.50 | 4.70 | 56 | 78.20 | 3.10 | 1.30 | -0.68, 3.28 | 45.02 |
| Czyzewski (2014) | 30 | 76.41 | 12.94 | 40 | 78.00 | 17.23 | -1.59 | -8.66, 5.48 | 20.65 |
| Kostro (2016) | 25 | 59.00 | 25.00 | 44 | 67.00 | 24.00 | -8.00 | -20.10, 4.10 | 9.68 |
| Pooled USMD (*I^2^*= 52.80%) | | | | | | | 1.21 | -2.98, 5.40 | 100.00 |
| **Quality of social interaction** | | | | | | | | | |
| Lee (2005) | 74 | 75.30 | 15.20 | 99 | 74.40 | 18.30 | 0.90 | -4.10, 5.90 | 27.05 |
| Okpechi (2013) | 26 | 72.00 | 5.30 | 56 | 74.00 | 3.10 | -2.00 | -4.19, 0.19 | 30.80 |
| Czyzewski (2014) | 30 | 85.13 | 11.27 | 40 | 72.00 | 15.96 | 13.13 | 6.75, 19.51 | 24.70 |
| Kostro (2016) | 25 | 69.00 | 23.00 | 44 | 72.00 | 20.00 | -3.00 | -13.78, 7.78 | 17.45 |
| Pooled USMD (*I^2^*= 84.80%) | | | | | | | 2.35 | -4.35, 9.04 | 100.00 |

PD, Peritoneal dialysis; HD, Hemodialysis, USMD, Unstandardized mean difference; 95%CI, 95% Confidence interval.

36

|  |  | |  | |  | |  | |  | |  | |  | |  | |
| --- | --- | --- | --- | --- | --- | --- | --- | --- | --- | --- | --- | --- | --- | --- | --- | --- |
|  |  |  |  |  |  |  |  |  |  |  |  |  |  |  |  |
|  |  |  |  |  |  |  |  |  |  |  |  |  |  |  |  |  |
|  |  |  |  |  |  |  |  |  |  |  |  |  |  |  |  |  |
|  |  |  |  |  |  |  |  |  |  |  |  |  |  |  |  |  |
|  |  |  |  |  |  |  |  |  |  |  |  |  |  |  |  |  |
|  |  |  |  |  |  |  |  |  |  |  |  |  |  |  |  |  |
|  |  |  |  |  |  |  |  |  |  |  |  |  |  |  |  |  |
|  |  |  |  |  |  |  |  |  |  |  |  |  |  |  |  |  |
|  |  |  |  |  |  |  |  |  |  |  |  |  |  |  |  |  |
|  |  |  |  |  |  |  |  |  |  |  |  |  |  |  |  |  |

Assessment of publication bias

|  |  | |  | | |  | |
| --- | --- | --- | --- | --- | --- | --- | --- |
|  |  |  |  |  |  |  |
|  | | | | | | | |
|  |  |  |  |  |  |  |  |
|  |  |  |  |  |  |  |  |
|  |  |  |  |  |  |  |  |
|  |  |  |  |  |  |  |  |
|  |  |  |  |  |  |  |  |
|  |  |  |  |  |  |  |  |
|  |  |  |  |  |  |  |  |
|  |  |  |  |  |  |  |  |
|  |  |  |  |  |  |  |  |
|  |  |  |  |  |  |  |  |
|  | | | | | | | |
|  |  |  |  |  |  |  |  |
|  |  |  |  |  |  |  |  |
|  | | | | | | | |
|  |  |  |  |  |  |  |  |
|  |  |  |  |  |  |  |  |
|  |  |  |  |  |  |  |  |
|  |  |  |  |  |  |  |  |
|  |  |  |  |  |  |  |  |
|  |  |  |  |  |  |  |  |
