# Supplementary material for: Comparisons of quality of life between patients underwent peritoneal dialysis and hemodialysis: a systematic review and meta-analysis
Source: Health Qual Life Outcomes. 2020 Jun 18;18:191. doi: 10.1186/s12955-020-01449-2 (PMC7302145; doi:10.1186/s12955-020-01449-2)

**Supplement Figures**

|  |  | Page |
| --- | --- | --- |
| Figure 1. | Funnel plot of SF-36 | 2 |
| Figure 2 | Contour funnel plot of SF-36 | 3 |
| Figure 3. | Funnel plot of EQ-5D | 4 |
| Figure 4. | Funnel plot of KDQOL | 5 |
|  |  |  |

Figure 1. Funnel plot of SF-36


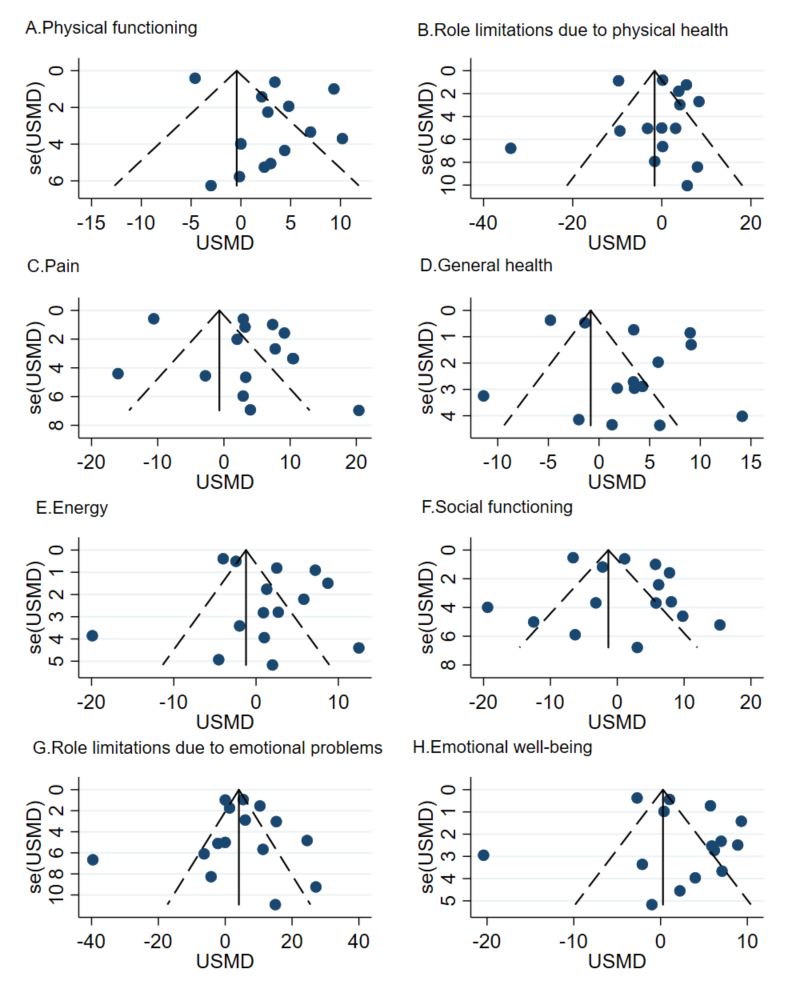


Figure 2. Contour funnel plot of SF-36


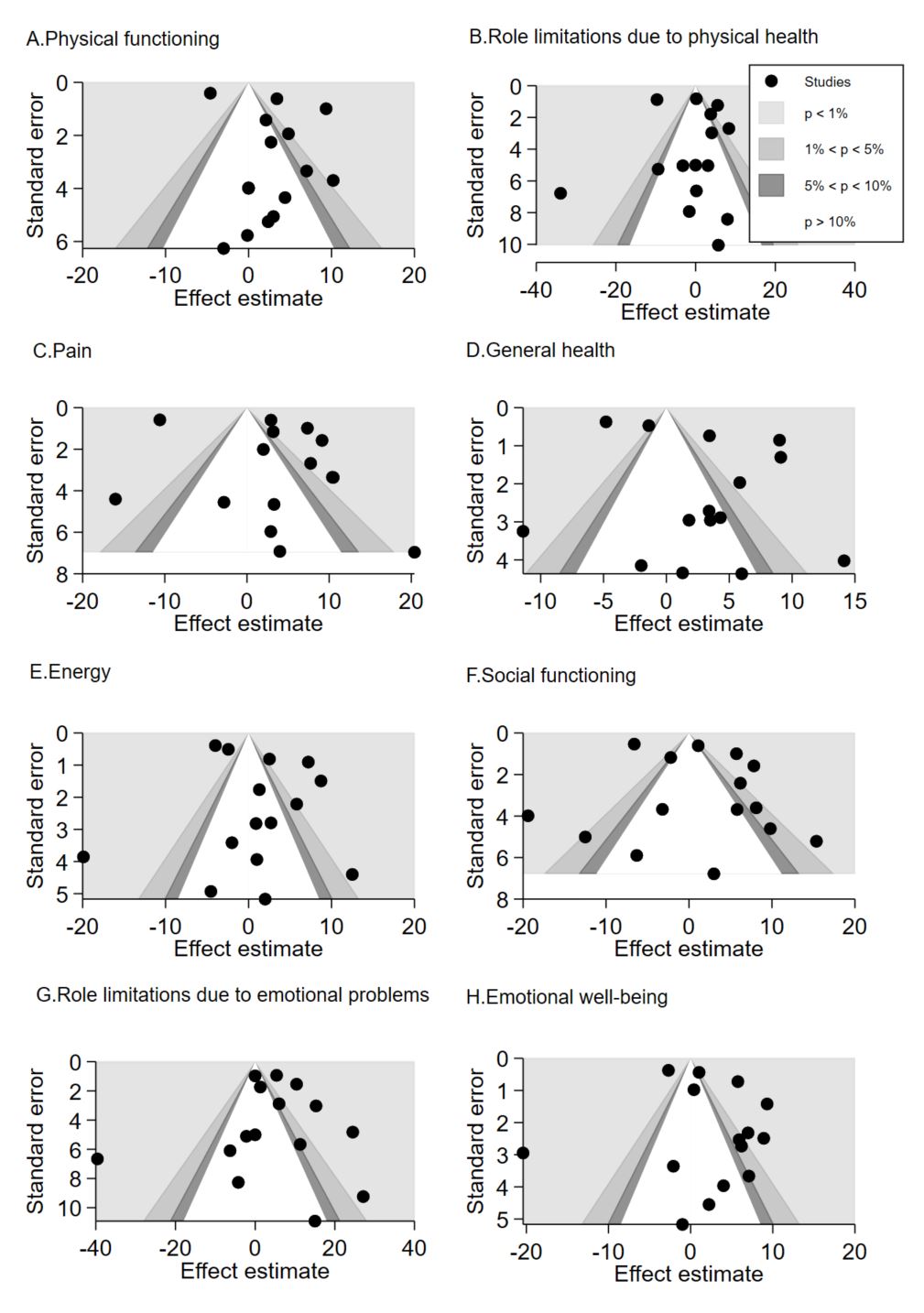


Figure 3. Funnel plot of EQ5D


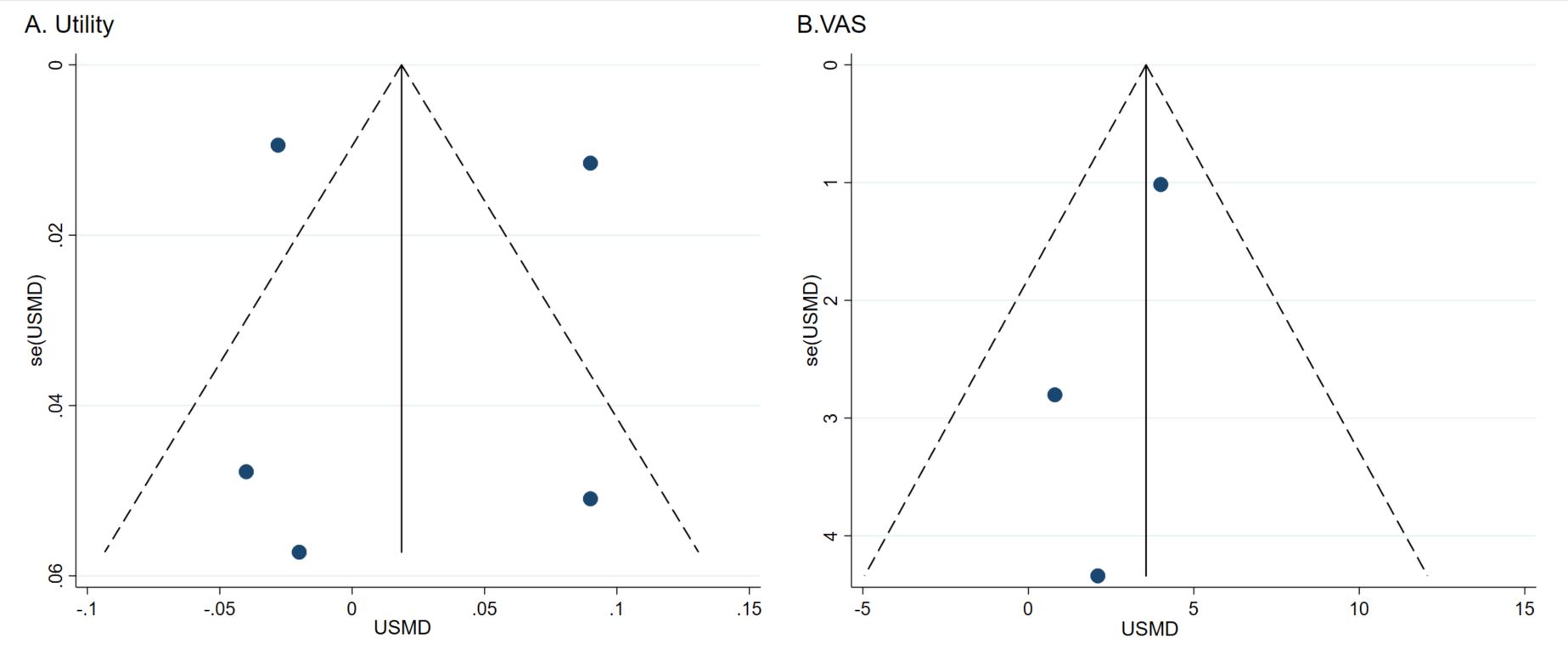


Figure 4. Funnel plot of KDQOL


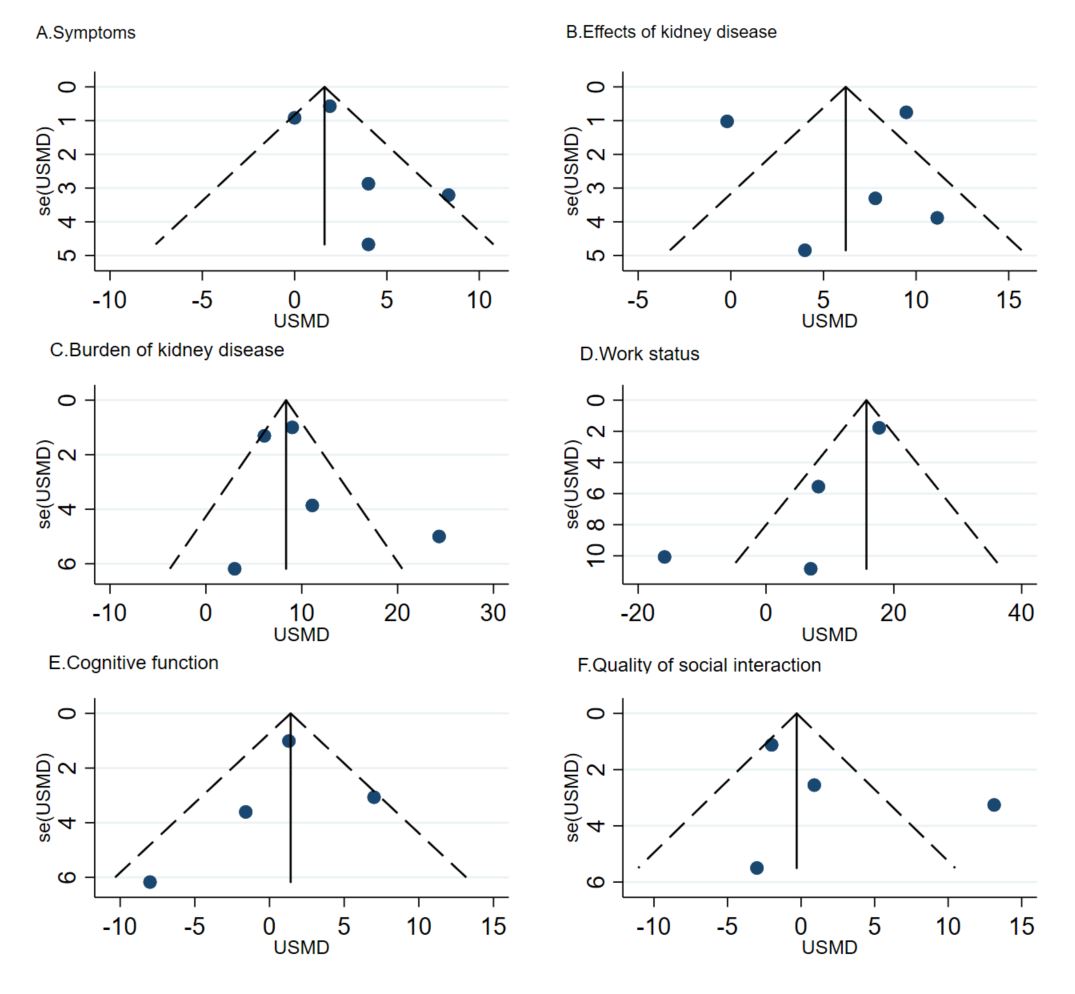

Supplement: Supplementary file 3 — Additional file 3. [file 12955_2020_1449_MOESM3_ESM.docx]
